# Histamine Recognition by Carbon Dots from Plastic Waste and Development of Cellular Imaging: Experimental and Theoretical Studies

**DOI:** 10.1007/s10895-023-03201-7

**Published:** 2023-03-28

**Authors:** Jessica M. Muro-Hidalgo, Iván J. Bazany-Rodríguez, José Guadalupe Hernández, Victor Manuel Luna Pabello, Pandiyan Thangarasu

**Affiliations:** 1 Facultad de Química, Universidad Nacional Autónoma de México (UNAM), Ciudad Universitaria, 04510 Mexico City, México; 2 Centro Tecnológico, Facultad de Estudios Superiores (FES-Aragón), State of Mexico, Universidad Nacional Autónoma de México (UNAM), 57130 Aragon, México

**Keywords:** Plastic waste, Carbon dots, Histamine, Fluorescence, Cu(II), Hg(II), and DFT

## Abstract

The present work highlights the sustainable approach for the transformation of plastic waste into fluorescent carbon dots (CDs) through carbonization and then they were functionalized with L-cysteine and o-phenylenediamine. CDs which were characterized by different analytical techniques such as X-ray diffraction (XRD), thermogravimetric analysis (TGA), Scanning electron microscopy (SEM), and transmission electron microscopy (TEM) are employed to recognize Cu^2+^, Fe^2+^, and Hg^2+^ ions. The results show that the fluorescence emission was considerably quenched, and it is consistent with the interference and Jobs plots. The detection limit was found to be 0.35µM for Cu(II), 1.38 µM for Hg(II), and 0.51µM Fe(III). The interaction of CDs with metal ions enhances the fluorescence intensity detecting histamine successfully. It shows that plastic waste-based CDs can be applied clinically to detect toxic metals and biomolecules. Moreover, the system was employed to develop the cellular images using Saccharomyces cerevisiae cells with the support of a confocal microscope. Furthermore, theoretical studies were performed for the naphthalene layer (AR) as a model for C-dots, then optimized its structure and analyzed by using the molecular orbital. The obtained TD-DFT spectra coincided with experimental spectra for CDs/M^2+^/histamine systems.

**Graphical Abstract:**

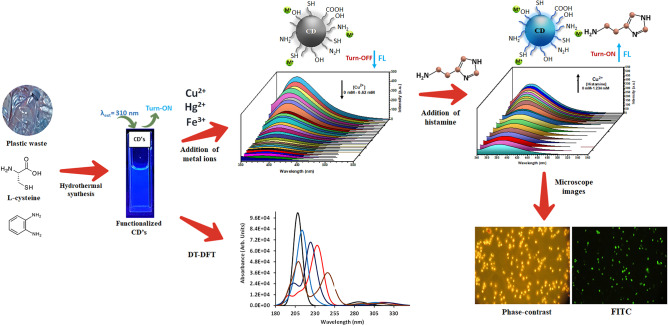

**Supplementary Information:**

The online version contains supplementary material available at 10.1007/s10895-023-03201-7.

## Introduction

Polymeric materials like polyethylene terephthalate (PET) are being used as industrial containers for the preservation of medicines, food, and soft drinks because of excellent physical and chemical properties such as high thermal stability, transparency, flexibility design, waterproof, good insulating character, low electrical conductivity, and inertness towards acids, sunlight, and microorganisms [1, 2]. It has been popular because of its low cost, easy to handle, lightweight, high strength, and long durability [3–5]. However, the accumulation of huge quantities of plastic waste in the environment is a serious issue [6] due to its non-degradable nature, contributing significantly to health and ecological imbalance [7–9]. Polymeric plastics contain a long carbon chain of about 62.6–92.2% [10] such as polyethylene (-C_2_H_4_)_n_, polypropylene (-C_3_H_6_)_n_, polystyrene (-C_8_H_8_)_n_, polyvinyl chloride (-C_2_H_3_Cl)_n,_ etc., [11, 12] and chemical recycling, or converting to gaseous, liquid, carbon-enriched materials are being considered [13, 14]. The incineration of PET is strictly prohibited as it consumes a lot of O_2_/air (14 m^3^ kg^− 1^), causing toxic- and greenhouse effects [15, 16]. The biodegradation of plastics by mealworms [17, 18], and the transformation of plastic- waste into adsorbents are also considered [19].

The conversion of PET waste to carbon-based nanomaterials (carbon nanotubes, graphene, and Carbon Dots (CDs)) turned out to be interesting [2, 20–24] as they exhibit good optical properties [25]. CDs (size, < 10 nm) can be used as bio-imaging agents, photo-catalyst, sensors, and solar cells [24, 26–29]. It has been found that CDs are somewhat superior to metal-based particles with respect to biocompatibility, and environmental friendliness [30, 31]. The covalent carbon skeleton also enhances the stability of CDs [32, 33] as it contains carbon, oxygen, and nitrogen with a mixture of sp^2^/sp^3^ carbon lattices. If CDs were functionalized properly, water-soluble CDs can be obtained [16, 31, 34]. Chemo-sensors have been widely applied for the detection of multiple heavy metals and their sensitivity can be improved by increasing the large surface/volume ratio after considering a high degree of functionalization [35, 36].

The formation of CDs from polymer wastes is attractive and can attribute to the different degrees of carbonization [21]; however, these types of studies are limited in the literature. So, the present work deals with the eco-friendly CDs-based chemo-sensor for the detection of metal ions, emerging as an alternative technique [37–39]. Interestingly, the generation of CDs from plastic waste is considered to be an eco-friendly method as the CDs are formed from biomass carbon [40], peanut shells [41], orange juice [42], Jinhua bergamot [43], and lotus root [44]. It is known that CDs have been used in different applications such as in the interaction with different heavy metals, namely Cu^2+^ ions [45–47]; in the logic gate operation [48]; in the detection of ascorbic acid, phosphatase [49], glyphosate [50] or glutathione [46]; also in the monitoring of pesticide [51]; in the development of cell image/Cu^2+^ [37]. In addition, polyamine-functionalized carbon quantum dots also have been used for the determination of copper ions [52]. Furthermore, CDs have been doped with other materials such as N-co-doped CDs [53], N, S-CDs [54], S-CDs [55], CDs/Ag [56], Hg^2+^ [57] to improve their efficiency for the recognition of Hg^2+^ [58–60].

The presence of toxic metals such as copper in environmental samples affects drastically biological functions [61], causing oxidative stress that produces disorders related to diseases like Menkes, Wilson’s, Parkinson’s, and Alzheimer’s. Controlling copper content in water is challenging although there are several methods like atomic absorption spectrometry (AAS) [62–64], inductively coupled plasma atomic emission spectroscopy (ICP-MS) [65–67], GC-MS, LC-MS or electrochemical analysis [68] are being employed to determine the concentration of metal ions. However, these techniques are usually time-consuming due to the complicated sample preparation, complex sample processing, and expensive instrumentation. In the present work, we report the plastic waste-based fluorescent carbon dots (FCDs) to recognize Fe^3+^, Cu^2+,^ and Hg^2+^ ions along with histamine, which has been observed in spoiled food and used often as an indicator for food safety; therefore, the development of a rapid and sensitive method for the detection of histamine is essential. Thus, the present sequential system has been applied to develop the cellular images using *Saccharomyces cerevisiae* ATCC 9763 cells with the help of confocal microscope. The CDs were characterized appropriately by different analytical techniques such as XRD, FT-IR, TGA, SEM, and TEM. The intensity of fluorescence was quenched for the metal ions while for histamine, it was enhanced. Furthermore, the theoretical studies were performed for a single nanographene layer (**AR)** which has been considered a model for C-dots and studied the interaction of CDs with metal ions and histamine. To the best of our knowledge, there is no report on the performance of the functionalized CDs derived from plastic wastes for the detection of metal ions performing as a logic gate for the recognition of histamine. DFT was used to probe the function of CDs/M^n+^/histamine in the absorption spectra.

## Materials and Methods

### Materials

Chemicals and solvents (Sigma Aldrich) were purchased and used without further purification. Plastic bottles were collected from the waste cabbages and were recycled/ transformed into CDs.

### Synthesis of CDs

**Scheme 1:**
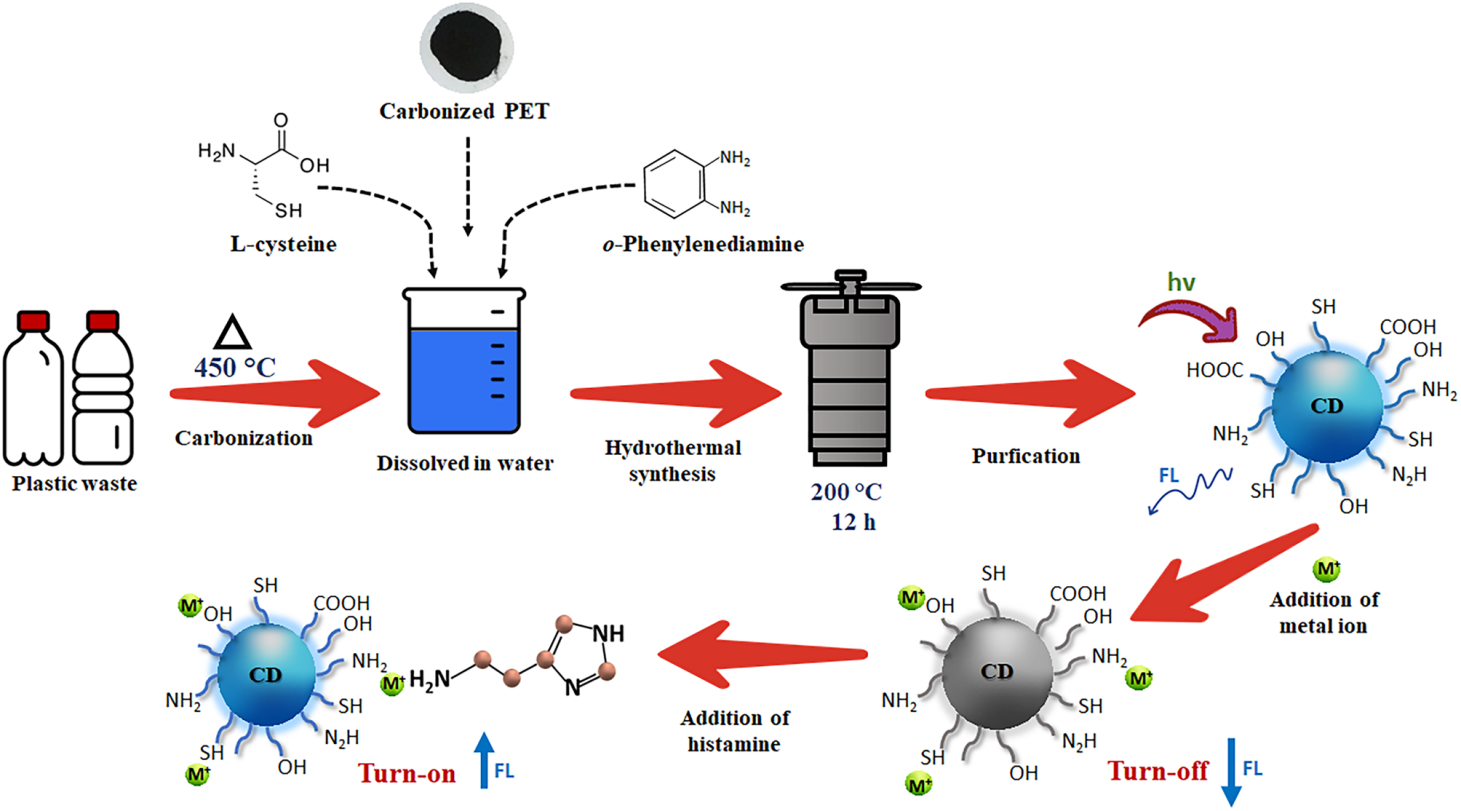
Synthesis of CDs from plastic waste

PET bottles were sized into small pieces (~ 1.0 × 1.0 cm^2^) and placed in a ceramic crucible to heat at 400 °C for 2 h in air. A dark brown product formed was crushed to a solid (PET-C). The obtained product (0.25 g) was mixed with L-cysteine (0.5 g) and *o*-phenylenediamine (0.25 g) and dissolved in de-ionized water (30 mL). The resulting mixture was transferred to a Teflon-contained autoclave and heated at 200 °C for 8 h. A brownish-black residue obtained was centrifugated (9000 RPM, for 20 min), filtered, and the filtrate was dried at 80 °C for 4 h to obtain the CDs, (Scheme 1).

For CDs samples, the XRD was performed on a Bruker D8 Advance Davinci, using Ni filter for Cu Kα radiation (λ = 1.541Å), and the diffraction angle at *2θ* was in the range of 20° to 80° operating at a voltage of 40 kV, 30 mA. The crystallite size of CDs was calculated by using Scherer’s formula. The morphology and size of the CDs were analyzed by scanning electron microscopy (SEM, JEOL JSM-5900-LV). The nature of CDs was studied by transmittance electron microscopy (TEM, JEOL JEM-2010, resolution: 3.0 nm HV), equipped with an EDX detector, fully automated on all 5 axes. Infrared spectra (4000 cm^− 1^ to 600 cm^− 1^, Perkin-Elmer Spectrum 400 FTIR/FIR) were recorded for CD_S_ to determine the functional groups like -C = O, -OH, and -NH_2_. The thermal stability of samples was studied by thermogravimetric analysis (TGA METTLER TOLEDO DSC1) in alumina cuvettes; airflow: 50 mL/min for each sample (10 ± 1 mg) with a precision of ± 0.02 °C ± 0.2 °C; heating range: 50 to 900 °C. For CDs samples, the fluorescence properties were studied on F-96 Pro and the absorption spectra were measured on Perkin-Elmer Lambda 25.

### Recognition Studies

First, metal nitrate solution (10 mM) of different cations such as Al^3+^, Ca^2+^, Cd^2+^, Co^3+^, Cu^2+^, Fe^3+^, Hg^2+^, Ni^2+^, K^+^, Mg^2+^, Ba^2+^, Na^+^ and Zn^2+^ was prepared. The metal binding studies were performed by adding aliquots of stock solutions of the respective metal ions to the CDs solution (0.016 ppm), which was buffered with an aqueous solution of HEPES (20 mM at pH = 7.0). The final concentration of [M]_final_ was 20 µM. The intensity of fluorescence emission of the sample was recorded at 387 nm. The titration of CDs with metal ions was also carried out by adding aliquots of stock solutions (analytes: Fe^3+^, Cu^2+^, Hg^2+^) to CDs (0.016 ppm) which were buffered as indicated above. The whole mixture solution was allowed to equilibrate for 2 min at room temperature and then recorded the emission spectra at 310 nm using a 10 mm quartz cuvette. For each successive addition of metal ions (Cu^2+^, Hg^2+,^ or Fe^3+^ (0 mM to 0.62 mM) to the CD solution (0.016 ppm), the decrease of the intensity was analyzed. The stoichiometry of the receptor CDs with the metal ions (especially with Cu^2+^) was determined by Job’s plot. The different proportions of CDs: Cu^2+^ was prepared as follows: 1:9, 2:8, 3:7, 4:6, 5:5, 6:4, 7:3, 8:2, and 9:1, and then measured their fluorescence intensities which were then plotted against the concentration as (I-I_o_)·X vs. X (X=[CDs ]/[CDs ]+[M]).

The limit of detection (LOD) for CDs to Cu^2+^, Hg^2+,^ or Fe^3+^ was determined from the plot (fluorescence intensity vs. the concentration of the metal ion). The fluorescence quantum yield was also calculated using the equation:

$${{\Phi }}_{fs}={{\Phi }}_{fr}\left(\frac{{L}_{s}}{{L}_{r}}\right)\left(\frac{{A}_{s}}{{A}_{r}}\right){\left(\frac{{n}_{s}}{{n}_{r}}\right)}^{2}$$

Ф_fs_ = quantum yields of sample; Ф_fr_ = quantum yields of reference sample; A_s_ = absorbance of sample and A_r_ = absorbance of the reference; L_s_ and L_r_ = lengths of the absorption cells; n_s_ and n_r_ = refractive indices of the sample and reference solutions, respectively [69]. PL quantum yield (Ф_fr_) was measured using quinine sulfate in sulfuric acid solution (0.05 M) as a reference (literature quantum yield was 0.54 at 310 nm n_r_ = 1.33) as a standard for carbon dots. In order to minimize re-absorption effects, the fluorescence cuvette was kept under 0.05 at the excitation wavelength [70].

### Cellular Imaging

The potential for cellular imaging of the CDs was tested by using *Saccharomyces cerevisiae* ATCC 9763 cells. Yeast cells were suspended in 20 mL of distilled water to which CDs were dispersed (0.016 ppm). Yeast cells were smeared on a microscope slide and treated for 10 min with CDs dispersion (25 µL of 0.016 ppm). Finally, the samples were observed at an epi-fluorescent microscope (Carl Zeiss AXIO Scope A1).

## Results and Discussion

### Characterization

**Fig. 1:**
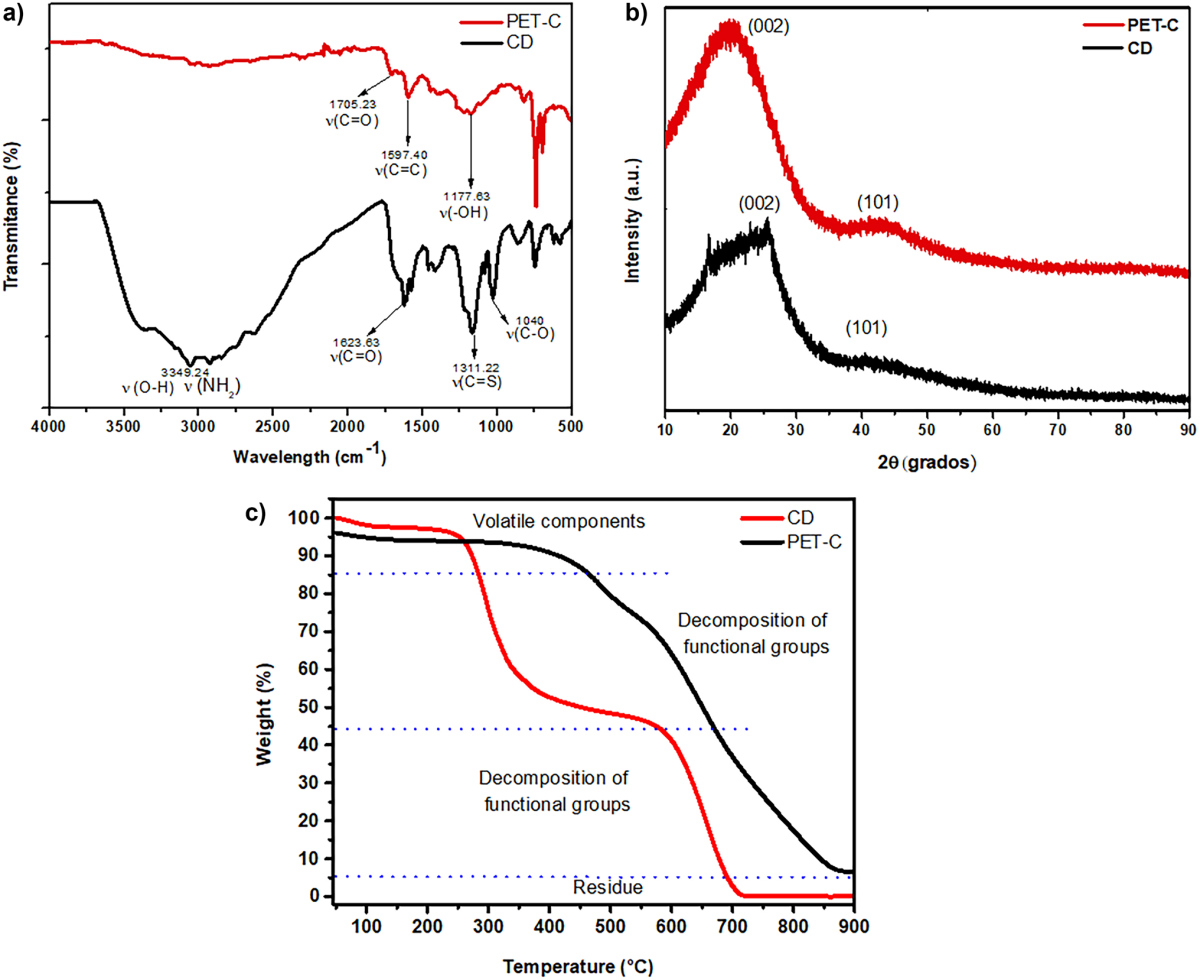
Analytical characterization of CDs: **a** FTIR, **b** XRD; **c** TGA analysis

FT-IR, XRD, and TGA: CDs are derived from the plastic contain mostly C, H components and do not have any hetero atoms (functional groups); however, CDs (from *o*-phenylenediamine and L-cysteine) might have -NH_2_, -OH, -C-C- and -C-H groups. Therefore, we have used IR technique to observe the signals corresponding to -C-C-, -C-H, -NH_2_ and -OH bonds presenting in the CDs. In the FT-IR spectra (Fig. 1a), it has been observed the signals corresponding to O–H and N–H (3349 cm^–1^), C–H (2890 cm^–1^), C–O (1623 cm^–1^), C = C (1575 cm^–1^), C – N (1416 cm^–1^ ) and C – O (1214 cm^–1^) [71]. The peaks of S-H (2633 cm ^–1^), C = S (1311 cm^–1^), C – O (1003 cm^–1^), C = C (1597 cm ^–1^), and C = O (1705 cm^–1^). The results show that the surface of CDs was enriched with hydroxyl, carbonyl, amino, and thiol groups that support the water solubility of the sample. XRD was performed for CDs and noticed a broad peak at 26° with the prominent 2θ value, corresponding to the (002), and the size of the particle was D = 0.39 nm, corresponding to abounding sp^3^ defects of carbon-based materials. The appearance of the characteristic peak has coincided with those reported in JCPDS 41-1487 (graphite) [72] (Fig. 1b). This means that the interlayer spacing (around 0.39 nm) is approximately agreed with that of the graphite 002 crystal plane (0.34 nm). CDs have a wider interplanar spacing, which may be caused by the doping of heteroatoms (such as N and S) which generally increase the repulsive force between the layer of the molecules. The electronegativity of N is larger than that of C, but the atomic radius is smaller than C. The crystalline CDs (Fig. 1b) were found to be triclinic types [16]. In contrast, the plastic waste that was carbonized (PET-C) shows a sharp diffraction peak at approximately 21° with a center of 2θ, as can be seen from the XRD peak shape that the PET-C is presented a crystalline state. The average grain size (D) was calculated by Scherrer’s formula [73].

$$D=\frac{0.9{\uplambda } }{Bcos\theta }$$

where λ is 0.15406 nm, θ is the Bragg angle, and B is the full width at half maximum.

The thermal performance of the sample was studied by the TGA technique (Fig. 1c) and the results show that CDs are significantly stable up to 100 °C. In the plot, two prominent bending of weight loss caused by the moisture was observed and it is also contributed by the pyrolysis of the functional groups from the CDs, and it is consistent with the weight loss around 250 and 575 °C [74]. This means that the weight loss was observed at stepping temperature after 100 °C as there is water evaporation and the burning of both carbon surface and carbon core structures occurred around 400 °C [75].

**Fig. 2:**
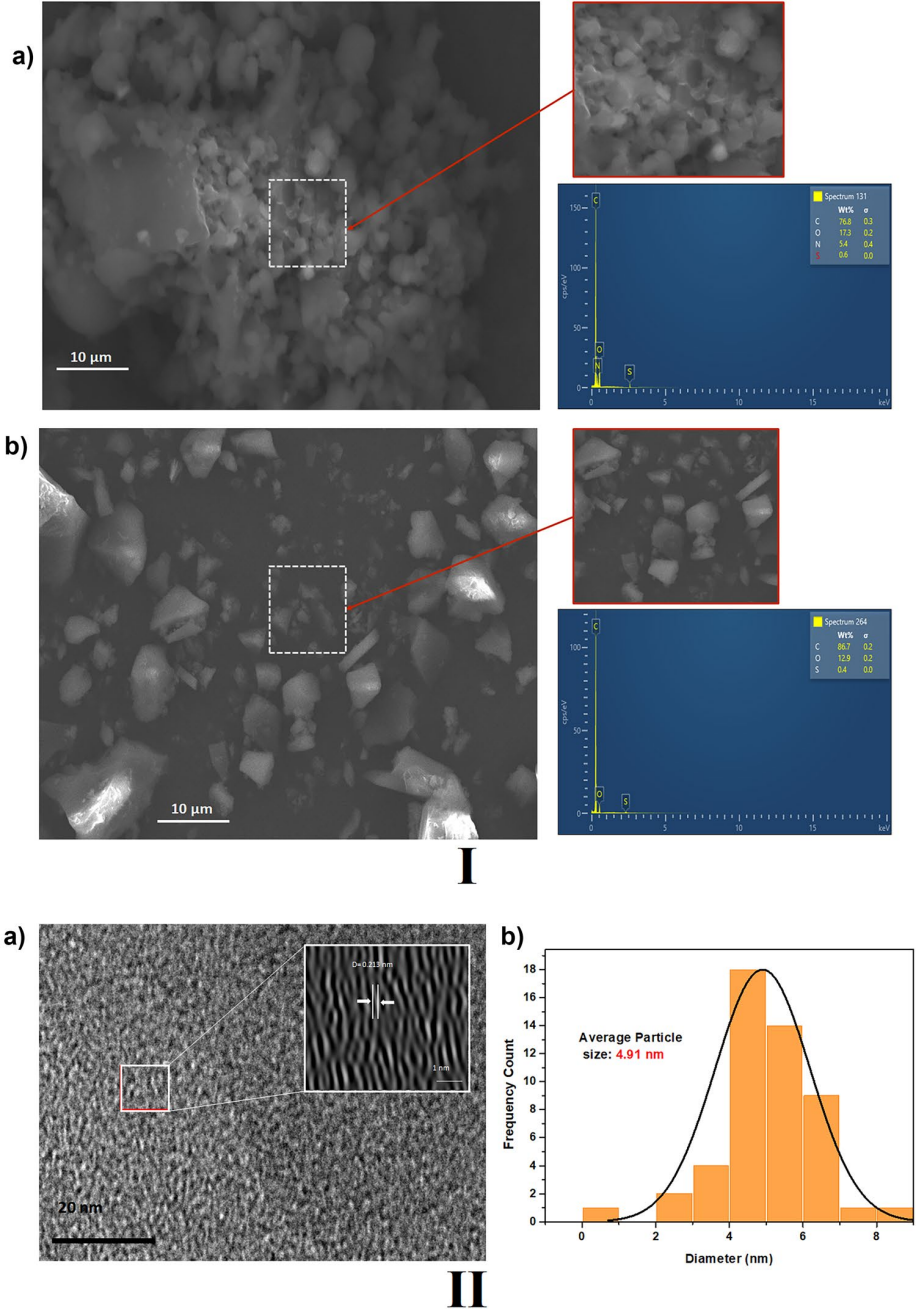
**I** SEM and EDS: (a) CDs and (b) PET-C.; **II)** TEM images: (a) CDs and (b) average particle size

The SEM was recorded for CDs and PET-C (Fig. 2Ia-b) and determined the morphology of particles. The CDs present a regular morphology in spherical and porous shapes, in addition to uniformity. Mostly, CDs are agglomerated in the form of clusters, showing that the size of CDs is much smaller than that of the PET-C which is presented in a porous/irregular shape with little uniformity (there is no agglomeration). The dimension of the CD is one of the most important properties because this allows a greater area of ​​contact with the contaminants and therefore their interaction, the PET-C not having a nanometric size prevents it from being used as a chemo sensor as well as its low affinity with other elements and particles. EDS was used to estimate the elemental composition for CDs: C (77.5%), O (16.5%), S (5.5%), and N (0.6); for PET-C: C (78.1%), O (20.5%) and S (1.4%), which verifies the presence of N and S derived from the addition of L-cysteine ​​and o-phenylenediamine to the synthesis to improve its characteristics. For both cases, Carbon is the element with the highest mass percentage present in nanomaterials.

Transmission electron microscopy (TEM) imaging as described provides information about the structure, phases, and orientations of the sample, whether it is amorphous or crystalline. TEM was used to characterize the microstructures of the CDs. The material showed uniform dispersion and spherical particles between 3 and 10 nm, with average diameters of approximately 4.91 nm (Fig. 2IIa-b). TEM images showed that the CDs had an average lattice spacing of approximately 0.213 nm well interplanar spacing; when the molecules are located in adjacent parallel planes and each of these planes has a designated Miller index, these indices are made up of three numbers which indicate the coordinates of a vector in three dimensions (x, y, z), corresponding to the (0 0 2) planes of graphite, which when compared to the clear, strong, broad X-ray diffraction (XRD) peak centered at around 26° indicated that the CDs had a graphitic structure, indicating the good crystallinity of CDs.

### Metal Binding Analysis

UV-Vis absorption and fluorescence emission (PL) spectra were performed for the CDs in an aqueous solution, presenting pale yellow under visible light, and a bright blue color under fluorescence illumination (310 nm). The results show an absorption peak at 280 nm (Suppl. Mat. Fig. S1.), corresponding to n-π* and π-π* transitions from C = C and C = O, presenting in CDs [76], and determined minimum bandgap energy [77, 78]. Fluorescence emission was observed after excitation at 310 nm with high intensity at 392 nm, showing that the conjugated π-electrons are cross-linked with different functional groups through which it has enhanced the fluorescence emission [76]. Table 1 enlists the different CDs originated from different carbon sources and their photoluminescence properties and kind of detection ion. The quantum yield was measured to be 31.81% and it was estimated using quinine sulfate as a theoretical standard [79]. The result is excellent as compared to the carbon dots prepared from other polymers (Table 2). High quantum yields resulted for our FCDs were due to the high doping of nitrogen and sulfur into the CDs, leading to the formation of favorable emissive states.

**Table 1:** The quantum yield of samples using quinine sulfate as a reference

**Precursor**	**Quantum yield** **(**${\varvec{\Phi }}_{\varvec{f}\varvec{s}}$**)**	**Reference**
Quinine sulfate	0.540 (known)	[79]
Plastic cups	0.590	[80]
Waste plastic bottle of PET	0.057	[81]
Waste plastic bags	0.039	[82]
Waste expanded polystyrene	0.200	[16]
Polyurethane	0.330	[83]

**Table 2:** Different carbon sources for CDs and the photoluminescence properties and detection

**Source of carbon dots**	**Abs. wavelength (nm)**	**Emission (nm)**	**Detection of cations**	**Reference**
Waste plastic bags	320	438	Fe^3+^	[82]
Waste polyethylene terephthalate	340	434	Fe^3+^	[81]
Perfluorooctane sulphonate	320	447	Fe^3+^	[84]
Red lentils	360	448	Fe^3+^	[85]
Egg white	315	420	Fe^3+^	[86]
Tomato	360	450	Fe^3+^	[87]
Papaya peel	370	450	Fe^3+^	[88]
Pronus cerasifa fruits	360	450	Fe^3+^	[89]
Sweet potato	360	442	Fe^3+^	[90]
Cranberry beans	380	450	Fe^3+^	[91]
Rise residue	360	440	Fe^3+^	[92]
Corn bract	406	408,670	Hg^2+^	[93]
Glutathione	371	442	Hg^2+^	[53]
Orange juice and ethylenediamine	360	449	Hg^2+^	[42]
Pomelo peel	365	444	Hg^2+^	[94]
Citric acid anhydrous and ethenyldiamine	360	438, 352	Hg^2+^	[58]
Green tea, ascorbic acid	365	385	Hg^2+^	[95]
Citric acid and sodium sulfide	345	445	Hg^2+^	[55]
Lotus root	375	435	Hg^2+^	[44]
Hair	330	415	Hg^2+^	[96]
Sodium citrate And polyacrylamide	330	451	Cu^2+^	[97]
Ethanediamine (EDA) and glacial acetic acid	365	454	Cu^2+^	[98]
Sulfuric acid and nitric acid	360	580	Cu^2+^	[49]
Peanuts shells	312	413	Cu^2+^	[99]
Leek	360	440	Cu^2+^	[40]
Pear juice	360	455	Cu^2+^	[41]
Waste polyolefins residue	490	540	Cu^2+^	[100]
Carbon Dots Functionalized (CDs)	310	392	Cu^2+^, Hg^2+^, Fe^3+^	Present work

**Scheme 2:**
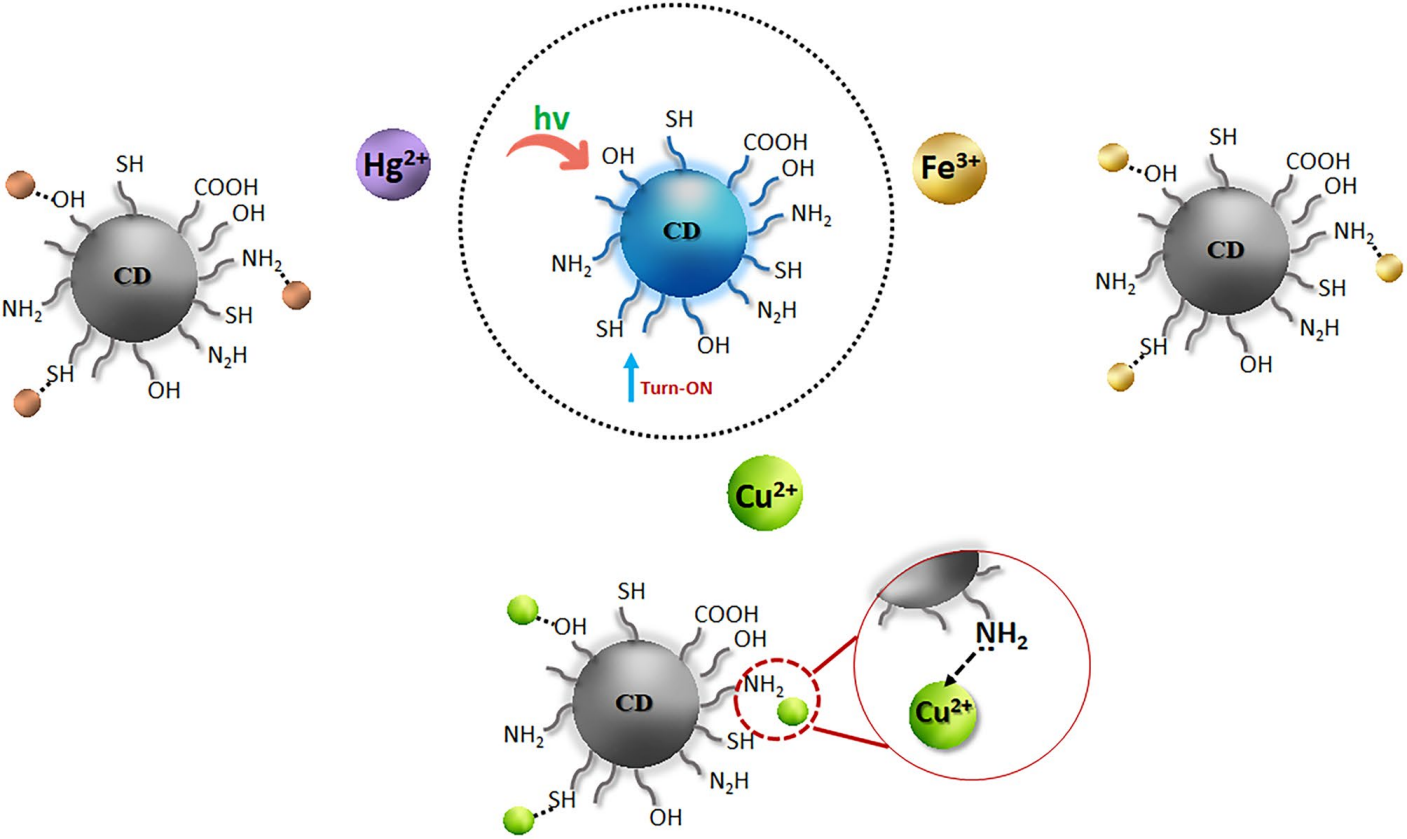
The quenching of fluorescence of CDs with Hg^2+^, Cu^2+,^ and Fe^3+^ ions

The metal binding analysis of CDs was explored by adding aliquots of stock solutions of the respective cation as nitrate salt (Al^3+^, Ca^2+^, Cd^2+^, Co^3+^, Cu^2+^, Fe^3+^, Hg^2+^, Ni^2+^, K^+^, Mg^2+^, Ba^2+^, Na^+^, and Zn^2+^), final concentration of [M]_final_ was 20 µM, after buffering with aqueous HEPES (20 mM at pH = 7.0) of CDs (0.016 ppm) (Suppl. Mat. Fig. S2). For the mixture solution, the fluorescence intensity measured at 387 nm was plotted against the respective cations (Na^+^, K^+^, Mg^2+^, Ca^2+^, Ba^2+^, Al^3+^, Co^3+,^ and Zn^2+^). Notably, the addition of hard paramagnetic metal ions such as Ni^2+^ has resulted in a modest decrease in emission intensity, but it was still significantly lower than that observed for Fe^3+^ (I_0_/I_F_ = 8.12) as well for Cu^2+^ (I_0_/I_F_ = 10.30). On the other hand, the addition of a soft diamagnetic metal ion, namely, Cd^2+^ gave a small quenching in the intensity, and yet it was considerably lower than that detected for Hg^2+^ (I_0_/I_F_ = 7.68). In general, the presence of -NH_2_ and -SH groups in CDs increases their affinity toward soft ions such as Cu and Hg as compared to hard metal ions. It means that the existence of paramagnetic centers favors the quenching of CDs as compared to the diamagnetic metal ions. After analyzing the results, it was noticed a significant quenching in the fluorescence intensity for Cu^2+^, Hg^2+,^ and Fe^3+^, attributing to paramagnetic or heavy atoms and it influences the photo-induced electron transfer (PET) (Scheme 2).

Furthermore, the spectral titration was performed for Cu^2+^ and Hg^2+^ with CDs (0.016 ppm) in an aqueous solution and it was also seen a quenching of emission with the increasing concentration of Cu^2+^ or Hg^2+^ (Suppl. Mat. Fig. S2). The intensity of the fluorescent emission was measured for each successive addition of Cu^2+^ (10 mM), decreasing considerably for M = Cu^2+^, Hg^2+^, and Fe^3+^ (0 mM to 0.62 mM). The plot was drawn for the concentration with the fluorescence intensity giving good linearity.

LOD is found to be 0.35 µM (R^2^ = 0.997) for Cu^2+^; 1.058 µM (R^2^ = 0.989) for Hg^2+^ and 0.510 µM (R^2^ = 0.993) for Fe^3+^. Using the 3σ method, the limit of detection (LOD) for CDs was determined as follows:

$$\mathrm{Limit}\;\mathrm{of}\;\mathrm{Detection}\left(\mathrm{LOD}\right)=\frac{3\sigma}{m_{cc}}$$

$$\sigma=\mathrm{Standard}\;\mathrm{deviation}\;\mathrm{of}\;\mathrm{triplicate}\;\mathrm{experiments}$$

$$m_{cc}=\mathrm{Slope}\;\mathrm{of}\;\mathrm{the}\;\mathrm{calibration}\;\mathrm{curve}$$

### Job’s Plot

**Fig. 3:**
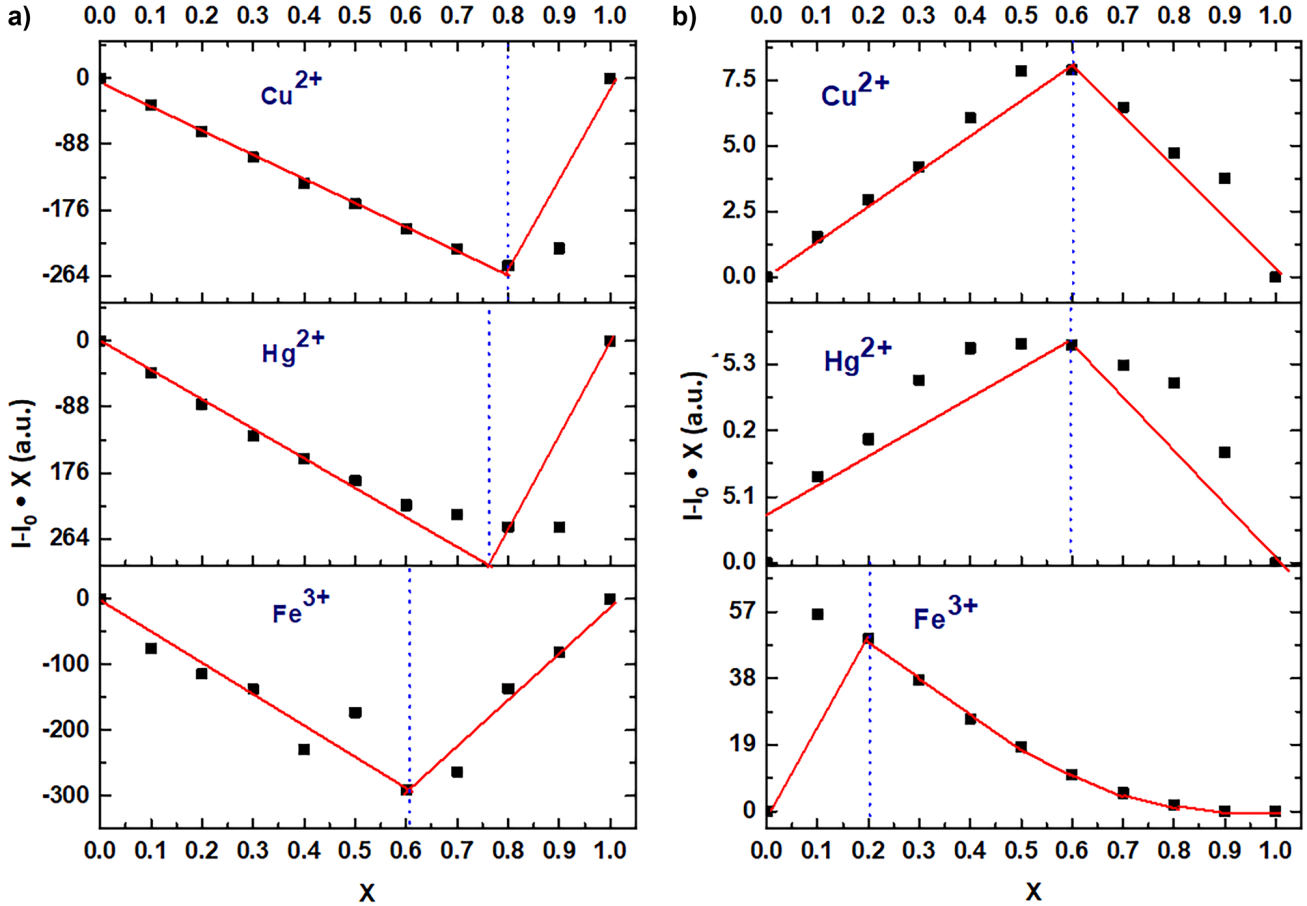
**a** Job´s plot stoichiometry analysis: CDs vs. M^n+^ at pH. 7; and **b** Stoichiometry analysis at pH 7: His vs. CDs-M^n+^. X (X=[CDs ]/[CDs ]+[M])

The stoichiometric ratio of FCDs with M = Cu^2+^, Hg^2+^, Fe^3+^ was analyzed through a Jobs plot (Fig. 3). A solution of metal ion solution and CDs (0.016 ppm) was prepared and maintained the stoichiometry concentration (0 ppm to 100 ppm) for metal ions and 100 ppm to 0 ppm for CDs and the volume of solution in the cell was 2.5 mL. The ratio for CDs (80 ppm) with Cu^2+^ (20 ppm) was 0.8:0.2; for CDs (76 ppm) with Hg^2+^(24 ppm) was 0.76:0.24; and for CDs (60 ppm) with Fe^3+^ (40 ppm) was as 0.6:0.4.

### Turn-on Histamine Detection: Turn-on Histamine Detection

**Fig. 4:**
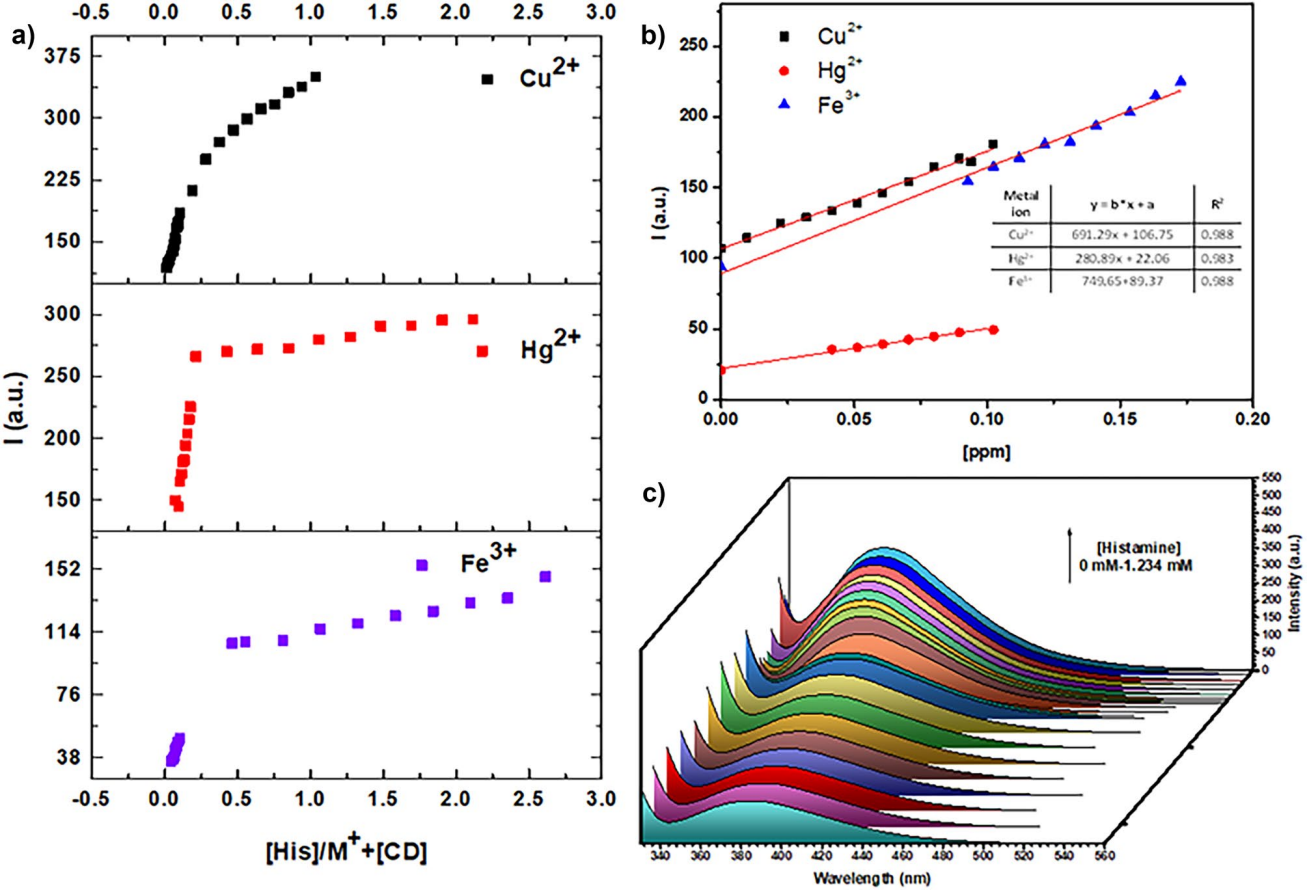
**a** Fluorescence intensity vs. concentration of histamine + metal ions; **b** The linear relationship of the intensity with the concentration of metal ions; **c** Fluorescence spectral titration against the concentration of histamine (0-1.234 mM) in the presence of Cu^2+^ and CDs

As a first step, the relative selectivity of the CDs-M^n+^(Cu^2+^, Fe^3+^, Hg^2+^) towards diamines was analyzed using histamine, ethylendiamine, cadaverine, putrescine, spermine, spermidine, dimethyl-1,3-propanediamine and dimethylethylenediamine ([Diamine]final = 0.01mM). The diamines were added to a buffered aqueous solution of CDs-M^n+^(0.6437 ppm) and measured the emission intensity. The results show an enhancement in the intensity for the CDs-M^n+^ as shown in Suppl. Mat. Figure 8. The fluorescence intensity was exceptionally low for the CDs-M^n+^ with the addition of diamine such as cadaverine, putrescine, spermine, spermidine and dimethyl-1,3-propanediamine. For the addition of ethylendiamine and dimethylethylenediamine has exhibited a modest enhancement of the emission intensity, but it was still significantly lower than that observed for histamine. So, the fluorescence detection of histamine was performed by using CDs having metal ions (Cu^2+^, Fe^3+^ or Hg^2+^), showing a turn-on fluorescence with respect to the concentration of histamine (Scheme 2, Fig. 4a-c). It is established a best linear fit for histamine (0-1.23mM) (see Job’s plot, Table S1). The limit of histamine detection for each metal ion was: 0.193 µM for Cu^2+^, 0.36 µM for Hg^2+,^ and 2.76 µM for Fe^3+^. In the presence of histamine, the fluorescent CDs can be recovered because of the strong interaction between metal ions (Cu^2+^, Fe^3+^ or Hg^2+^) and histamine. In this system (M^n+^-CDs), the functional groups on the surface of CDs (carboxylates, amines, thiols, hydroxyls) serve as the recognition sites and it supports to the binding of amino group with imidazole from histamine in order to form a coordination complex that effectively restores the fluorescence of CDs. The coordination complex of histamine with Cu^2+^, Fe^3+^ or Hg^2+^ is thermodynamically stable due to the chelate effect generated from histamine which acts a chelating ligand, showing a high affinity for a metal ion than the analogous monodentate ligands.

The Job’s plot for histamine with CDs having Cu^2+^, Hg^2+,^ or Fe^3+^ was determined (Fig. 3b), showing that with the increased concentration of histamine (0-150 µM), the FL intensity for CDs was increased (see Table S2).

### Logic Gate Systems for CDs

**Scheme 3:**
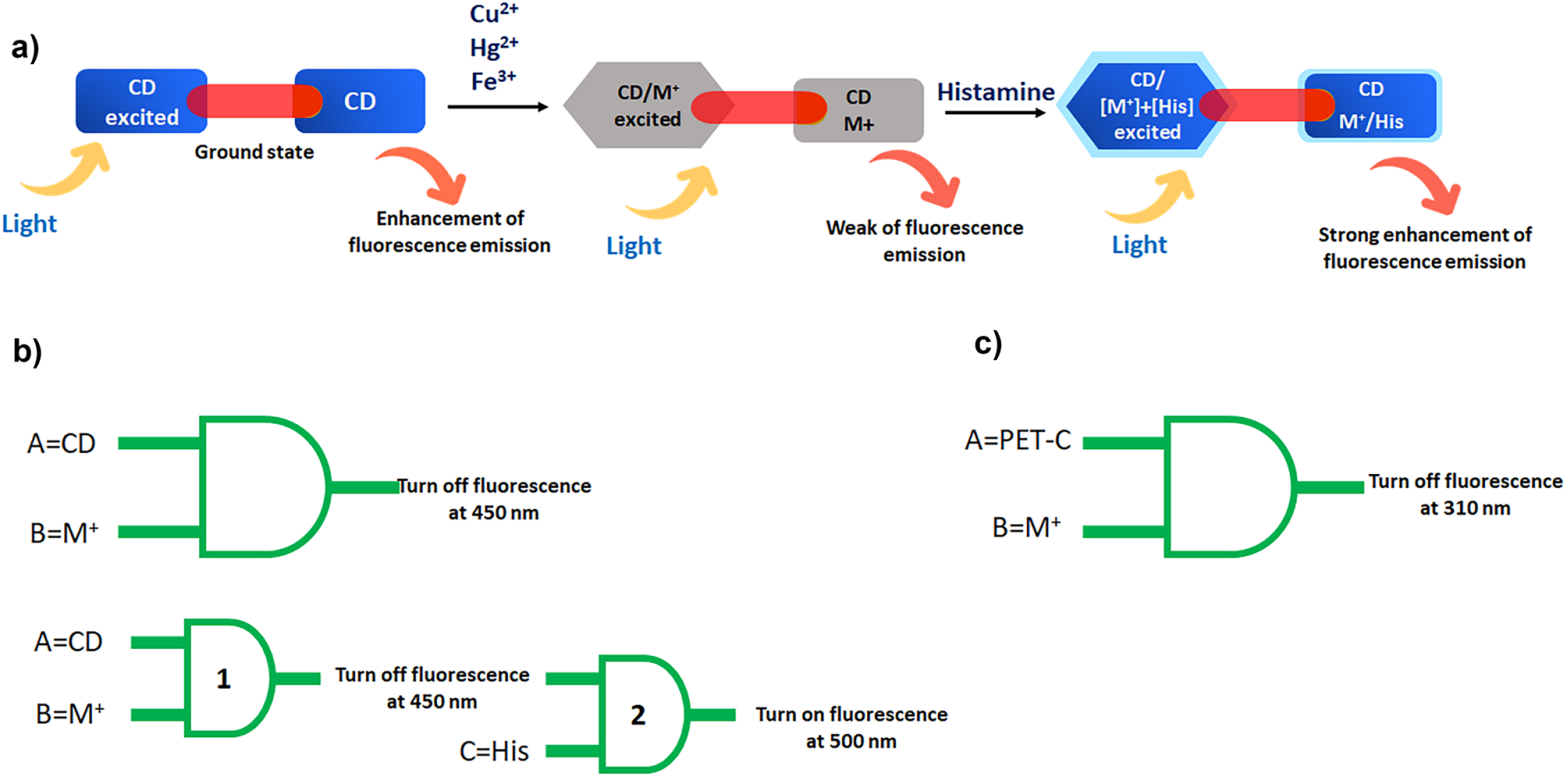
The color change of fluorescence emission: **a** CD = Turn on neon blue; **b** CD/M^+^= weak blue; **c** CD/ M^+^+His = strong enhancement blue fluorescence emission after addition of histamine to CD/M^+^

Logic gates for CDs are important for detecting various analytes (cations, anions, amino acids, pesticides, antioxidants, etc.), and generally most of the logic gates are fundamentally based on fluorescence spectroscopy because of their sensitiveness. After analyzing the present results, it was found that there exists a logic gate system in the recognition of histamine in the presence of metal ions (Cu^2+^, Hg^2+^, Fe^3+^). This means, the enhancement of fluorescence intensity for histamine with CDs was seen only if the metal ions are presented in sequential order; however, the intensity was low or absent if we mixed other combinations of CDs with histamine. First, an AND logic gate was performed through the binding of CD with metal ions [A = 1, B = 0], turning off the fluorescence was seen at 450 nm. Nevertheless, the fluorescence intensity was increased if (other AND logic gate) in the order of CD/Cu^2+^, CD/Hg^2+^, CD/Fe^3+^ as an input (in the presence of histamine). This indicates that CDs first recognize the metal ions, and then detect histamine (Scheme 3).

The enhancement of fluorescence intensity has occurred only when all inputs [A = 1, B = 1, C = 1] are given, as it functions as a three inputs AND logic gate system. But, for other combinations with negative inputs [A = 0, B = 0, or C = 0], no fluorescence was exhibited (Table 3).

**Table 3:** Logic gates of different systems

**Typical two-input and logic gate**				
**A** **(CD)**	B (M+)		Output (Fluorescence at 450 nm)	
**1**	0		1	
**0**	1		0	
**0**	0		0	
**1**	1		0	

**Three input logic gates**				
**A** **(CD)**	B (M+)	C (His)		Output (Fluorescence at 390 nm)
**1**	0	0		1
**0**	1	0		0
**0**	0	1		0
**1**	0	1		1
**0**	1	1		0
**1**	1	1		1

## Cellular Imaging

### Yeast Imaging with CDs

**Fig. 5:**
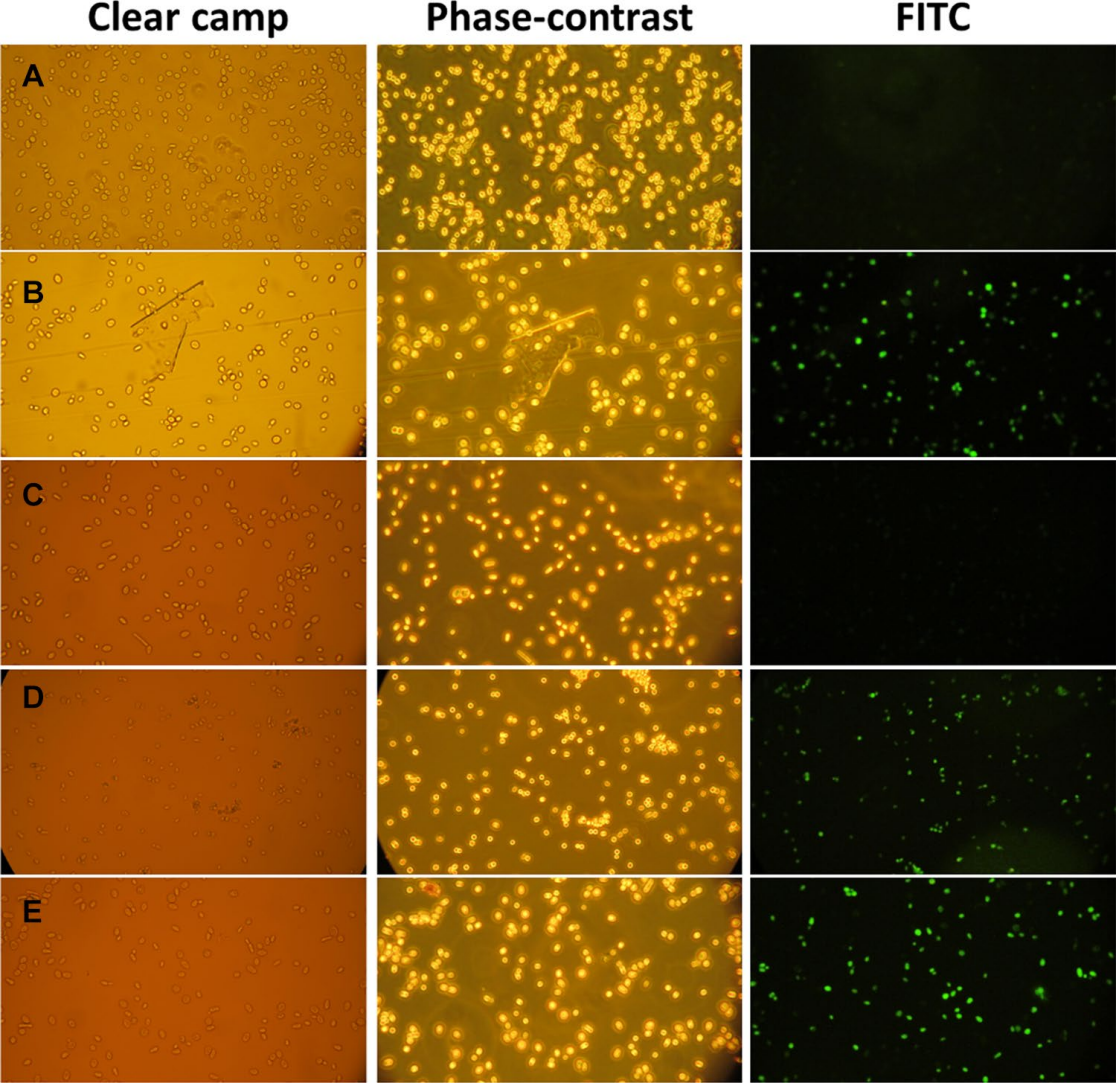
Microscope images: **A** row yeast cells, **B** row cells with CDs, **C** row cells with Cu(II)-CDs, **D** row cells with Cu(II)-CDs-Histamine, and **E** row cells with histamine. (Left column) clear camp microscopic images, (Center column) phase-contrast microscopic images, and (Right column) microscopic images with a fluorescence filter of FITC

*Saccharomyces cerevisiae* ATCC 9763 cells were observed by epi-fluorescent microscope and recorded the microscopic images of yeast cells with CDs (0.016 ppm) (Fig. 5, left column; clear camp microscopic images, column center; phase-contrast microscopic images, and column right; microscopic images with a fluorescence filter of FITC). Row A corresponds to the yeast control sample without CDs, and B row represents the yeast cells with CDs, which demonstrated the suitability of CDs serving as a potential alternative fluorescence probe for the cell imaging.

### Yeast Cell Imaging with Cu(II)-CDs and Histamine

*The image of* yeast cells were also observed in the presence of Cu(II)-CDs and the fluorescence intensity corresponding to CDs is quenched because of copper(II) as it is a paramagnetic ion (Fig. 5, C row). In the yeast cell imaging with Cu(II)-CDs and histamine, the cell images were observed in the presence of Cu(II)-CDs having histamine. It can be seen that in the presence of histamine, the fluorescent CDs can be recovered due to the existence of a strong interaction between Cu(II) and histamine. For Cu(II)-CDs, the functional groups (-COOH, -NH_2_, -SH) on the surface of CDs perform as active sites forming a metal complex through amino group and imidazole from histamine; thus, it regenerates effectively the fluorescence intensity (see Fig. 5, D row). The E Row corresponds to CDs with histamine, showing that histamine does not modify the fluorescence from CDs.

## Theoretical Studies

### Theoretical Model

**Fig. 6:**
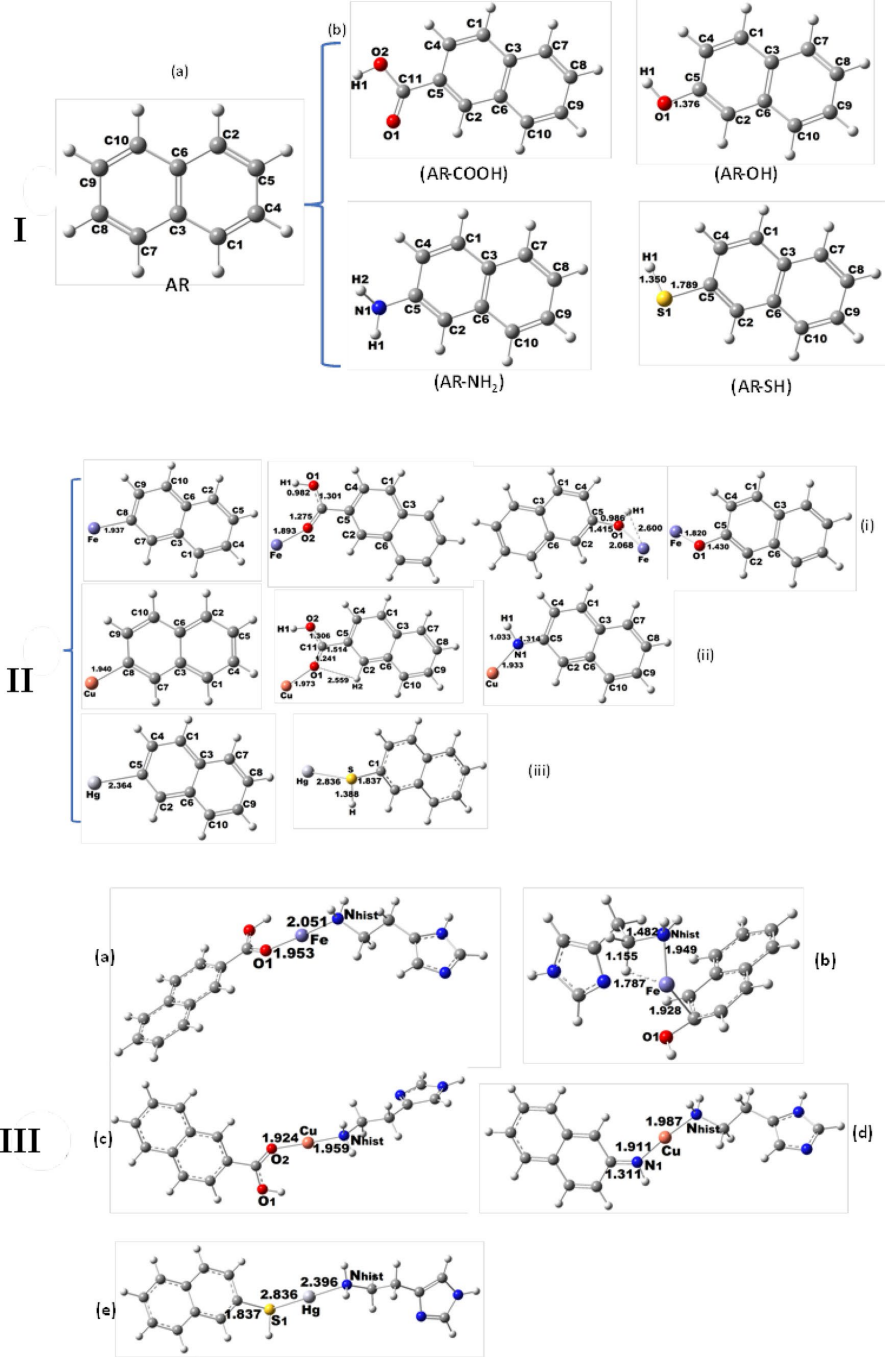
**I**). Optimized molecular structure: (a) **AR** represents C-dots and (b) C-dots with functional groups; AR-COOH, AR-NH_2_, AR-OH, and AR–SH. **II**). Optimized geometries for C-dots which interacted with the metal ions; i): **AR**-Fe^3+^, **AR**-HCOO–Fe^3+^, **AR**-HO–Fe^3+^, **AR**-C–O–Fe^3+^ ii): **AR**-Cu^2+^, **AR**_−_HCOO–Cu^2+^, **AR**_−_HN–Cu^2+^, and iii): **AR**_−_Hg^2+^, **AR**_−_SH–Hg^2+^, **III**) Optimized geometry of **AR** with metal ions (Fe^3+^, Cu^2+,^ and Hg^2+^) + histamine: a)AR-COOH-Fe-histamine; b)**AR**-OH-Fe-histamine, c) **AR**-COOH-Cu-histamine, d) **AR**-NH_2_-Cu-histamine, and e) **AR**-SH-Hg-histamine.

Structural optimization of naphthalene (**AR**) as a model for Carbon dots was studied by the DFT using Gaussian 09 having B3LYP and analyzed the influence of different functional groups (-COOH, OH, NH_2_) in the electronic and geometrical properties (Fig. 6(I)a and 6(I)b)0. The interaction of **AR** (other substituents such as -COOH, -OH, -NH_2_, and -SH) with metal ions (Fe^3+^, Cu^2+,^ and Hg^2+^) was investigated as indicated previously [101], and derived TD-DFT spectra for the above compounds [102–104] at the DGDZVP basis set [101] .

In the interaction of metal ions with molecule AR forms bond Fe-C (AR) (1.937 Å), Fe-O(COOH) (1.893 Å), Fe-O(OH) (2.068 Å), Fe-H(OH) (1.600 Å) and Fe = O (1.820 Å). Similarly, for Cu –C (**AR**) (1.940 Å), C-O(COOH) (1.937 Å), Cu-N(NH_2_) (1.933 Å) and N-C (**AR**) (1.314 Å) and for Hg-C(A) (2.364 Å), Hg-S (2.836 Å) and H – C(AR) (2.437 Å) were obtained (Fig. 5(II)). The results show that the bond length of carbon with Fe^3+^ (1.937 Å) or with Cu^2+^ (1.940 Å) is relatively shorter than with Hg^2+^ (2.364 Å). In particular, the metal ions coordination with other functional groups attached to ligand (**AR**) studied was showing the formation of Cu^2+^-O(COOH) (1.973 Å), Cu^2+^-NH_2_ (1.933 Å) and Fe^3+^-O(COOH) (1.893 Å), (Fe^3+^-O(OH) (2.068 Å) and Hg^2+^-SH (2.836 Å). This is consistent with the molecular orbital studies where the overlapping of the orbital of **AR** with that of Fe^3+^ and Cu^2+^ ions was noticed (Supp. Mat. Fig. S9). In general, in the study, a significant change in the bond distance of M-AR (M = Fe^3+^, Cu^2+^, Hg^2+^) was observed if **AR** possesses different functional groups (**AR**-COOH, OH, NH_2_, SH) (Table S3). For example, the presence of a strong bond was seen for Fe^3+^ and Cu^2+^ with **AR** as compared to Hg^2+^, for which, the bond length was somewhat longer. Thus, **AR** with functional groups is suitable for the detection of Fe^3+^ and Cu^2+^ (Supp Mat. Fig. S10, Table S5).

**Fig. 7:**
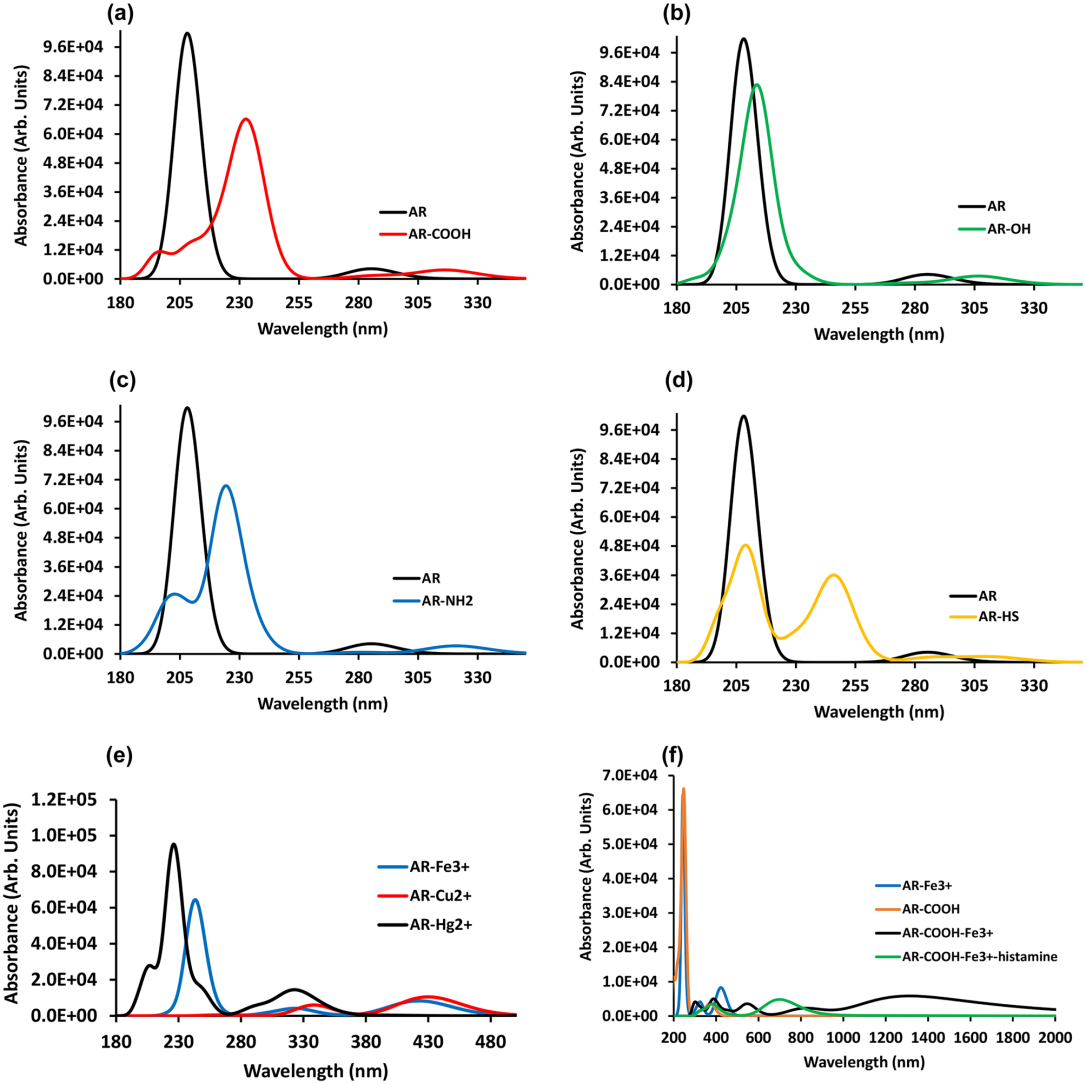
TD-DFT absorption spectra for C-dots: **a** without functional groups and AR-COOH; **b** with functional groups on the surface of C-dots and AR-OH), **c** without functional groups and **AR**-NH_2_; **d** without functional groups and **AR**-HS; and **e** for **AR** as C-dots with M^3+/2+^, M = Fe^3+^, Cu^2+^, and Hg^2+^) and (f) AR, COOH, Fe3 + and histamine group

Recently, the optical and electronic properties of the quantum dots based on graphene were reported [105]. Accordingly, we have proposed an adequate model that represents C-dots after considering a single nano-graphene layer; however, in a realistic model, several layers could be aggregated (Fig. 6I, II, III). The TD DFT absorption spectrum (Fig. 7) calculated for the above structures somewhat coincided with that observed for the C-dots which were prepared by hydrothermal method [106–110], in particular, the absorption spectral peaks (200–400 nm) have coincided approximately with the experimental values [108, 110]. The C-dots structures consist of amorphous carbon having functional groups during the carbonization of organic molecules [109].

In the spectra, the peak appeared approximately around 200–247 nm for **AR** having functional groups (Fig. 7) [111], representing two aromatic rings having several carbon atoms [112]; it means that the peak representing C-dots was observed for the π-π* transition [106, 109]. Thus, the chain of AR carbon rings is considered a model for C-dots (see Supp. Mat. Fig. S9). The electronic and aromatic properties of nanographene are highly dependent on the aromaticity of the rings [113, 114] and the particular peak can be shifted if the aromaticity of the Hückel rule is satisfied for aromatic rings attached with different functional groups. Several reports [109, 110, 115–117] describe carbon dots possess different functional groups on their surface; thus we have proposed the selected structures represent the carbon dots’ surface.

The influence of the functional groups in the absorption spectra was analyzed (Fig. 6a-d, and see Supp. Mat. Table S4), observing that the peak associated with π-π* transitions underwent a redshift. The visible absorption band is associated with the metal ion *d-d* electronic transition [106, 109]. The interaction of AR as C-dots with iron, copper, and mercury was studied as **AR** -Fe^3+^, **AR**-Cu^2+^, and **AR** -Hg^2+^ (Fig. 7e). The results show clearly the presence of three absorption peaks around 435 nm for **AR** -Fe^3+^, 431 nm for **AR**-Cu^2+^, and 329 nm for **AR** -Hg^2+^. After an analysis of their DOS (Fig. S7 (ii)), the transitions between *d-*occupied states and *s-*unoccupied states occurred, agreeing with a pure quadrupole transition selections rule *l* = ± 2, where *l* is the angular momentum quantum number [53]; as a result, these peaks 244, 266, and 214 nm were obtained. Similarly, the transitions between occupied bands (*d* and *s)* to unoccupied bands (*p)* can occur for the *l* = ± 1 dipole transition selection rule, but the peak can appear at lower wavelengths [50]. In the present work, the **AR** molecule as C-dots with different functional groups (COOH, NH_2,_ and HS) can interact with metal ions followed by histamine molecule; thus, in the structural calculation, a stable configuration of these adducts were obtained.

**Fig. 8:**
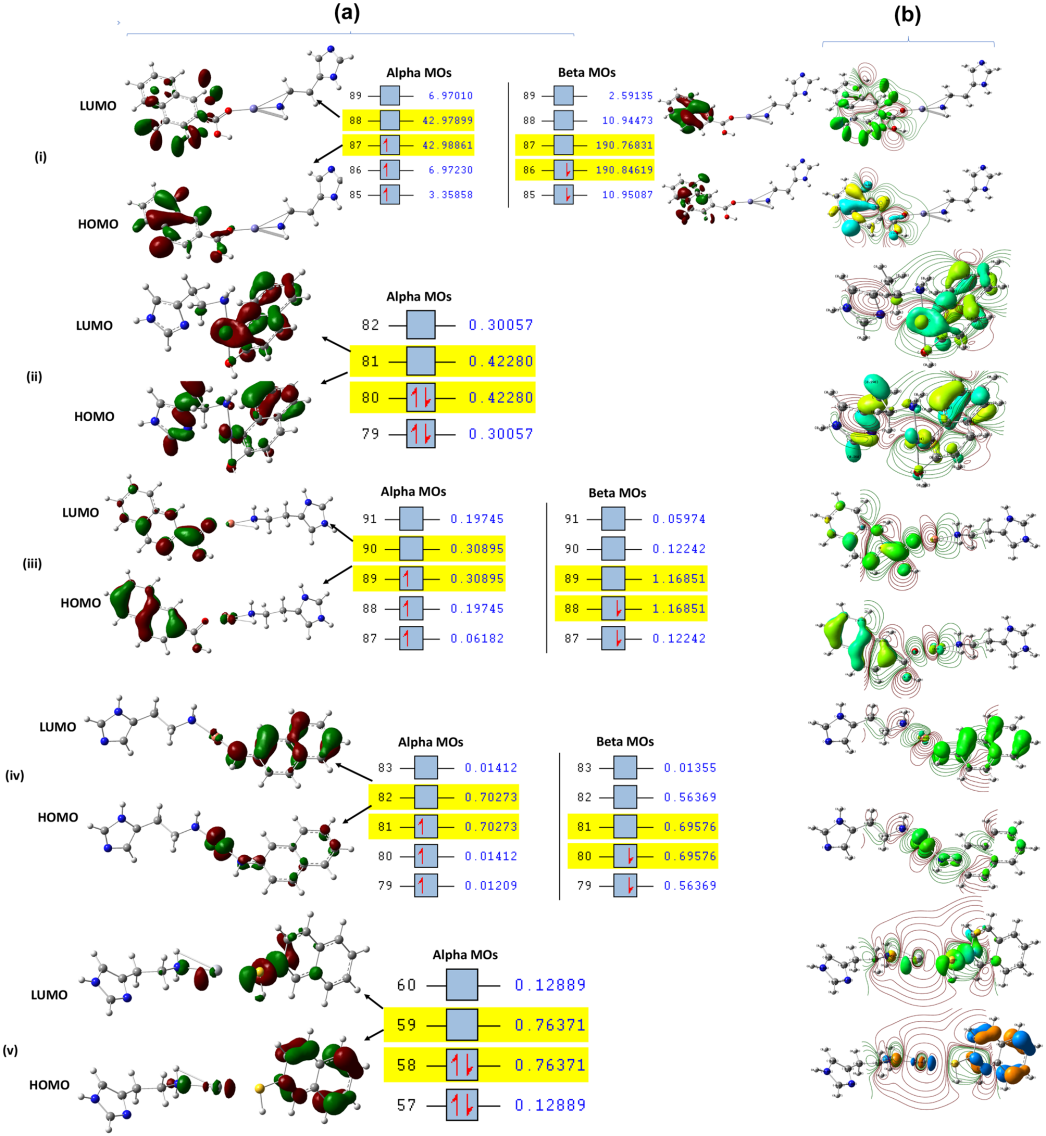
**a** NTOs, orbital 87, 80, 89, 81, 58 (particle), and orbital 88, 81, 90, 82, 59 (hole): **(i)** [**AR**-COOH-Fe^3+^-histamine], **(ii)** [**AR**-OH-Fe^3+^-histamine], **(iii)** [**AR**-COOH-Cu^2+^-histamine], **(iv)** [**AR**-NH_2_-Cu^2+^-histamine] and **(v)** [**AR**-SH-Hg^2+^-histamine], excited state number 17, 35, 30, 23, 35 at S = 1/2. **b** Electron density isosurface: **(i)** [**AR**-COOH-Fe^3+^-histamine], **(ii)** [**AR**-OH-Fe^3+^-histamine], **(iii)** [**AR**-COOH-Cu^2+^-histamine], **(iv)** [**AR**-NH_2_-Cu^2+^-histamine] and **(v)** [AR-SH-Hg^2+^-histamine] at gaseous state: the code of color for the structure C (grey), H (white), N (blue), O (red) and Fe (gray/lavender), Cu (red light) and Hg (gray light), and (contour 0.05 e Å−3), HOMO and LUMO: HOMO and LUMO contour plots (isosurface value = 0.05 au)

The adsorption energies and the bond distances (Mat. Supp, Table S5) for the interaction of CDs with Fe^3+^, Cu^2+^, and Hg^2+^ were obtained and they show that a stable bonding was seen for **AR**-COOH-Fe-N_histamine_ and **AR**-COOH-Cu-N_histamine_. However, for **AR** -SH-Hg, that interaction has not been stabilized adequately; but it has enough energy to replace the histamine molecule (2.396 Å) (Fig. 8a(iii)). According to the adsorption energy (1.106 eV), the exchange between histamine and C-dot is energetically favorable, suggesting that **AR**-COOH-Fe, **AR**-OH-Fe, **AR**-NH-Cu, **AR**-COOH-Cu, **AR**-SH-Hg/C-dots can interact favorably with histamine as a capping agent (dash-dotted line, see Supp. Mat. Fig. S10).

*Natural transition orbitals (NTOs)*: NTOs were obtained using DFT with B3LYP/DGDZVP at (S = 1/2) at the ground state for the following systems: [AR-COOH-Fe^3+^-histamine], [AR-OH-Fe^3+^-histamine], [AR-COOH-Cu^2+^-histamine], [AR-NH_2_-Cu^2+^-histamine] and [AR-SH-Hg^2+^-histamine] and determined appropriately the delocalization properties, especially for [AR-Fe^3+^], [AR-COOH], [AR-COOH-Fe^3+^], and [AR-COOH-Fe^3+^-histamine] [118]. The positive hole NTOs are localized on the H atoms and metal ions, and the electron NTOs are delocalized over the σ* orbital of the functional groups (COOH, NH_2_, OH, and SH, and N-histamine) or π* orbital of the **AR** moiety (Fig. 8a and see Supp. Mat. Figs. S10, S11, S12, S13 and S14). The TD-DFT absorption spectra obtained for the above systems show their electronic transitions [107, 108] (see Fig. 8). Furthermore, the transition of electron density between the ground and excited states reveals that the electron is removed from the occupied NTO (ground state) into the excited state (virtual NTO, particle density). After analyzing carefully several electronic states (~40) for all the systems, we have detected the electronic state which is able to exhibit relatively a large oscillator strength as number 17: (*f* = 0.0661, λ = 578.68 nm) for [**AR**-COOH-Fe^3+^-histamine], number 35 (*f* = 0.1901, λ = 241.58 nm) for [**AR**-OH-Fe^3+^-histamine]; number 30: (*f* = 0.2857, λ = 262.31 nm) for [**AR**-COOH-Cu^2+^-histamine], number 23: *f* = 0.1791 λ = 417.42 nm for [**AR**-NH_2_-Cu^2+^-histamine] and number 35 (*f* = 0.3958, λ = 222.55 nm) for [**AR**-SH-Hg^2+^-histamine]. So, the orbital transition is occurred through electronic state number (87, 81) HOMO to LUMO (88, 82) for α-orbital and HOMO (state number 86, 88, 80) to LUMO (87, 89, 81) for β-orbital, and it yields the absorption maximum (578.68, 417.42 nm) for [**AR**-COOH-Fe^3+^-histamine], and (871.39 nm) for [**AR**-NH_2_-Cu^2+^-histamine]. The orbital transition for [**AR**-COOH-Cu^2+^-histamine], [**AR**-OH-Fe^3+^-histamine], and [**AR**-SH-Hg^2+^-histamine] was seen at electronic states such as 80, 89, 58 (HOMO) to 81, 90, 59 (LUMO), giving absorption maximum of 241.58, 262.31, 222.55 nm, respectively. These transitions are assigned to an admixture of intraligand charge transfer (ILCT) and MLCT states (charge transfer).

The electron density movement was studied by an atom-connectivity method through a molecular graph (electronic contour density), which indicates the existence of the interaction between AR with metal ions. Because the electron density of − 1.014 for N1, -0.258 for C1, and a positive electron density of 0.442 for hydrogen, -0.531for O1 in [**AR**-COOH] was observed in the excited states for the system [**AR**-COOH-Fe^3+^] (Fig. 8bi). The interaction of AR-OH with Fe^3+^ at a perpendicular position was seen for [**AR**-OH-Fe^3+^] (see Fig. 8bii). A similar type of interaction was also observed for other systems such as [**AR**-COOH-Cu^2+^], **[AR**-NH_2_-Cu^2+^], [**AR**-COOH-Cu^2+^-histamine], [**AR**-NH_2_-Cu^2+^-histamine] and [**AR**-SH-Hg^2+^-histamine] in the excited state (Fig. 8biii, iv, and v). This is consistent with the electronic contour density analysis where the existence of interaction in [**AR**-COOH-Fe^3+^], [**AR**-OH-Fe^3+^], [**AR**-COOH-Cu^2+^], [**AR**-NH_2_-Cu^2+^], and [**AR**-SH-Hg^2+^] as well with the histamine was detected (Fig. 8ai-v). The same observation was seen for other remaining systems such as [**AR**-COOH-Fe^3+^-histamine], [**AR**-OH-Fe^3+^-histamine], [**AR**-COOH-Cu^2+^-histamine], [**AR**-NH_2_-Cu^2+^-histamine] and [**AR**-SH-Hg^2+^-histamine], (Hg^2+^) in the excited state.

## Conclusion

The transformation of plastic waste into fluorescent carbon dots (CDs) archived is characterized by different analytical methods, and then employed successfully for the recognition of Fe(III), Cu(II), or Hg(II). The results show that there is a distinct fluorescence emission caused by the interaction of the cations with CDs. It means that the fluorescence emission was turned off for the interaction of CDs with the above cations. The limit of detection was found to be as low as 0.35 µM for Cu(II), 1.38 µM for Hg(II), and 0.51 µM Fe(III). The CDs/M^2+^ system was then employed for sensing histamine exhibiting a “turned-on switch” and it enhances the fluorescence. It is consistent with the cell image development studies. The function CDs were analyzed by DFT using the naphthalene layer as a model for CDs and studied the interaction of metal ion detecting histamine and the obtained TD DFT spectra coincided with that of experimental spectra for CDs/M^2+^/histamine systems. It shows that plastic waste-based CDs can be used to detect toxic metals and histamine molecules.

### Electronic Supplementary Material

Below is the link to the electronic supplementary material.

Supplementary Material 1

## Data Availability

Not applicable.
