# Supplementary material for: Histamine Recognition by Carbon Dots from Plastic Waste and Development of Cellular Imaging: Experimental and Theoretical Studies
Source: J Fluoresc. 2023 Mar 28;33(5):2041–59. doi: 10.1007/s10895-023-03201-7 (PMC10539467; doi:10.1007/s10895-023-03201-7)
Supplement: Supplementary file 1 — Supplementary Material 1 [file 10895_2023_3201_MOESM1_ESM.docx]

**Logic gate understanding of histamine recognition by carbon dots from plastic waste and development of cellular imaging: Experimental and theoretical studies**

Jessica M. Muro-Hidalgo^1^, Iván J. Bazany-Rodríguez^1^, José Guadalupe Hernández^2^, Victor Manuel Luna Pabello and Pandiyan Thangarasu^1,*^

**Supplementary materials**

**Table S1.** Mass-volume relationships between CDs and Cu^2+^, Hg^2+^ and Fe^3+^.

| **Metal** | **Mass-volume relation** | |
| --- | --- | --- |
|  | **CD [ppm]** | **Metal [ppm]** |
| Cu^2+^  Hg^2+^  Fe^3+^ | 80  76  60 | 20  24  40 |

**Table S2.** Stoichiometry interaction between histamine, metal ions, and CDs.

|  | Stoichiometric ratio |
| --- | --- |
| His/Cu^2+^+CDs | 1:2 |
| His/ Fe^3+^ +CDs | 1:4 |
| His/ Hg^2+^ +CDs | 1:2 |

**Table S3**. Bond lengths (Å) and bond angles (°) resulted in different complexes with different functional groups using the Functional B3LYP/DGDZVP level of theory

| **Bond length (Å)** | **AR-Fe^3+^** | **AR-COOH-Fe^3+^** | **AR-OH-Fe^3+^** | **Bond length (Å)** | **AR-Cu** | **AR-COOH-Cu** | **AR-NH_2_-Cu** |
| --- | --- | --- | --- | --- | --- | --- | --- |
| Fe-C(COOH) | 1.937 |  |  | Cu-NH_2_ | 1.980 |  |  |
| Fe-O(COOH) |  | 1.893 |  | Cu-O(COOH) |  | 1.973 |  |
| Fe-OH |  |  | 2.068 | C-C(COOH) |  | 1.514 |  |
| C-C(COOH) |  | 1.481 |  | C-OH |  | 1.306 |  |
| C-OH |  | 1.301 | 1.415 | C=O |  | 1.241 |  |
| C=O |  | 1.275 |  | O-H |  | 0.983 |  |
| O-H |  | 0.982 | 0.986 | Cu=NH_2_ |  |  | 1.933 |
| **Bond Angle (°)** |  |  |  | C-NH |  |  | 1.314 |
| Fe-C=C | 118.2 |  |  | N-H |  |  | 1.033 |
| Fe-C-C | 123.2 | 154.4 |  | **Bond Angle (°)** |  |  |  |
| H-O-C |  | 116.9 | 108.7 | Cu-C=C | 117.2 |  |  |
| O-C-C |  | 114.0 | 117.6 | Cu-C-C | 121.0 |  |  |
| C-C=O |  | 121.9 | 120.1 | Cu-O-C |  | 145.9 |  |
| Fe-O-H |  |  | 111.7 | O=C-OH |  | 127.2 |  |
| Fe-O-C |  |  | 137.1 | HO-C-C |  | 111.9 |  |
| **Bond length (Å)** | AR-Hg^2+^ | AR-SH-Hg^2+^ | AR-S-Hg^2+^ | C-C=O |  | 120.8 |  |
| Hg-C | 2.364 |  | 1.805 | C-O-H |  | 115.5 |  |
| Hg-SH |  | 3.203 |  | Cu-NH=C |  |  | 141.6 |
| Hg-S |  |  | 3.098 | Cu-N-H |  |  | 107.3 |
| C-SH |  | 1.801 |  | HN=C-C |  |  | 121.2 |
| S-H |  | 1.391 |  |  |  |  |  |
| C5-HS |  | 2.437 |  |  |  |  |  |
| **Bond Angle (°)** |  |  |  |  |  |  |  |
| Hg-C=C | 119.4 |  |  |  |  |  |  |
| Hg-C-C | 118.8 |  |  |  |  |  |  |
| Hg-SH-C5 |  | 122.2 |  |  |  |  |  |
| Hg-S-H |  | 95.5 |  |  |  |  |  |
| C5-S-H |  | 98.7 |  |  |  |  |  |
| C4-C5-S |  | 118.0 | 120.4 |  |  |  |  |
| Hg-S-C5 |  |  | 107.6 |  |  |  |  |
| C2-C5-S |  |  | 120.5 |  |  |  |  |

**Table S4.** The wavelength of TD-DFT spectra of different C-dots with different functional groups and the heavy metal ions in the surface calculated at B3LYP functional with DGDZVP basis set at gaseous state.

|  |  | Wavelength (nm) | | | | | | |
| --- | --- | --- | --- | --- | --- | --- | --- | --- |
| Naphthalene **(AR)** | Compounds | λ1 | λ2 | λ3 | λ4 | λ5 | λ6 | λ7 |
|  | **AR** | 215 | 341 |  |  |  |  |  |
|  | **AR**-OH | 223 | 314 | 391 |  |  |  |  |
|  | **AR**-OH-Fe^3+^ | 330 | 411 | 753 | 1130 |  |  |  |
|  | **AR-**Fe^3+^ | 244 | 341 | 435 |  |  |  |  |
|  | **AR**-COOH | 208 | 249 | 367 |  |  |  |  |
|  | **AR**-COOH-Fe^3+^ | 313 | 398 | 552 | 816 | 1297 |  |  |
|  |  |  |  |  |  |  |  |  |
|  | **AR** | 216 | 325 |  |  |  |  |  |
|  | **AR**-COOH | 196 | 220 | 239 | 325 | 375 |  |  |
| **(AR)** | **AR-**NH_2_ | 213 | 237 | 317 | 385 |  |  |  |
|  | **AR**-Cu^2+^ | 266 | 343 | 431 |  |  |  |  |
|  | **AR**-COOH-Cu^2+^ | 198 | 207 | 233 | 255 | 306 | 410 | 611 |
|  | **AR**-NH_2_-Cu^2+^ | 297 | 341 | 398 | 522 | 771 |  |  |
|  |  |  |  |  |  |  |  |  |
|  | **AR** | 216 | 325 |  |  |  |  |  |
|  | **AR**-SH | 216 | 267 | 338 | 387 |  |  |  |
|  | **AR**-Hg^2+^ | 214 | 228 | 329 |  |  |  |  |
|  | **AR**-SH-Hg^2+^ | 184 | 200 | 235 | 273 | 321 | 456 | 704,1263 |
|  | **AR**-S-Hg^2+^ | 232 | 277 | 344 | 461 | 549 | 753 |  |

**Table S5.** Adsorption energies and bond distances for C-dots and molecules adsorbed on Fe^3+^, Cu^2+^ and Hg^2+^ ions.

| **System** | **Eads (eV)** | **Bond length (Å)** |
| --- | --- | --- |
| **AR**-Fe^3+^ | -45157.91 | Fe-C-dots (1.937) |
| **AR**-Cu_2_^+^ | -55485.39 | Cu-C-dots (1.98) |
| **AR**-Hg^2+^ | -5875.69 | Hg-C-dots (2.364) |
| **AR**-COOH-Fe^3+^ | -50349.32 | Fe-O(COOH) (1.893) |
| **AR**-OH-Fe^3+^ | -47240.22 | Fe-O(OH) (2.068) |
| **AR**-COOH-Cu^2+^ | -60675.76 | Cu-O(COOH) (1.973) |
| **AR**-NH_2_-Cu^2+^ | -57005.82 | Cu-N(NH2) (1.933) |
| **AR**-SH-Hg^2+^ | -6152.33 | Hg-S(SH) (3.203) |
| **AR**-S-Hg^2+^ | -224.53 | Hg-S (3.098) |
|  |  |  |
| Histamine | -9874.50 | C-N_hist_ (1.471) |
| **AR**-COOH-Fe^3+^-hist | -60229.51 | Fe-O1(1.953), Fe-N_hist_(2.051) |
| **AR**-OH-Fe^3+^-hist | -57152.17 | Fe-O1(1.954), Fe-N_hist_(2.041) |
| **AR**-COOH-Cu^2+^-hist | -70556.65 | Cu-NH (1.911), Cu-N_hist_(1.987) |
| **AR**-NH_2_-Cu^2+^-hist | -66887.66 | Cu-NH (1.911), Cu-H(NH) (2.461), Cu-N(Nhist) (1.987), Cu-H(NH_hist_) (2.496) |
| **AR**-SH-Hg^2+^-hist | -7888.91 | Hg-(N_hist_) (2.396), Hg-SH (2.836), Hg-H(NH_2_) (2.822, 2.846) |

**Table S6.** Electronic properties of the ligands (AR) with different functional groups (COOH, OH, NH_2_ and SH) and coordinated with different metals (Fe^3+^, Cu^2+^, and Hg^2+^)

| **Compounds** |  | **Homo** | **Lumo** | **ΔE** | **ɳ** | **σ** |
| --- | --- | --- | --- | --- | --- | --- |
| **AR** | -385.918 | -6.117 | -1.343 | 4.774 | 2.387 | 0.419 |
| **AR**-COOH | -574.527 | -6.451 | -2.019 | 4.431 | 2.216 | 0.451 |
| **AR**-OH | -461.152 | -5.883 | -1.328 | 4.554 | 2.277 | 0.439 |
| **AR**-NH2 | -441.282 | -5.541 | -1.127 | 4.413 | 2.207 | 0.453 |
| **AR**-SH | -784.075 | -6.003 | -1.481 | 4.523 | 2.261 | 0.442 |
| **AR**-Fe^3+^ | -1647.414 | -20.188 | -18.1523 | 2.036 | 1.018 | 0.982 |
| **AR**-COOH-Fe^3+^ | -1836.791 | -17.878 | -17.106 | 3.796 | 1.898 | 0.527 |
| **AR**-OH-Fe^3+^ | -1723.379 | -18.434 | -17.539 | 3.394 | 1.697 | 0.589 |
| **AR**-Cu^2+^ | -2024.173 | -20.647 | -18.891 | 1.756 | 0.878 | 1.139 |
| **AR**-COOH-Cu^2+^ | -2213.523 | -18.442 | -17.655 | 0.787 | 0.393 | 2.541 |
| **AR**-NH_2_-Cu^2+^ | -2079.639 | -19.978 | -18.110 | 1.868 | 0.934 | 1.071 |
| **AR**-Hg^2+^ | -214.352 | -6.022 | -2.836 | 3.186 | 1.593 | 0.628 |
| **AR**-SH-Hg^3+^ | -224.444 | -14.133 | -13.106 | 1.027 | 0.513 | 1.948 |

**Fig.S1. (**a) Photo-activity test (Synthesis o -phenylenediamine, L-cysteine and carbonized PET) in: UV-Vis (absorption), (b) Fluorescence (emission) to carbonized PET and Synthesis o -phenylenediamine, L-cysteine.


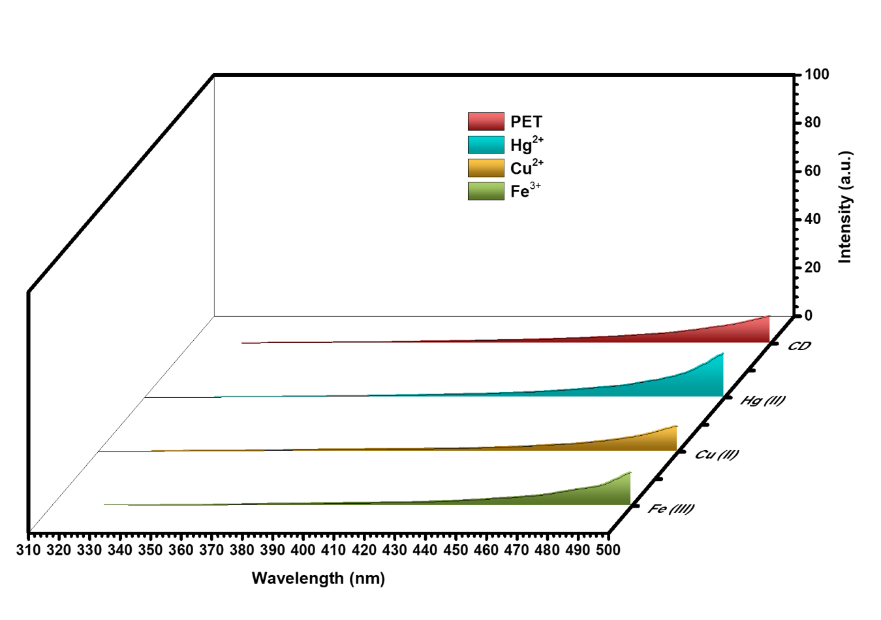


**Fig. S2.** Metal binding test (MBT) for PET-C.


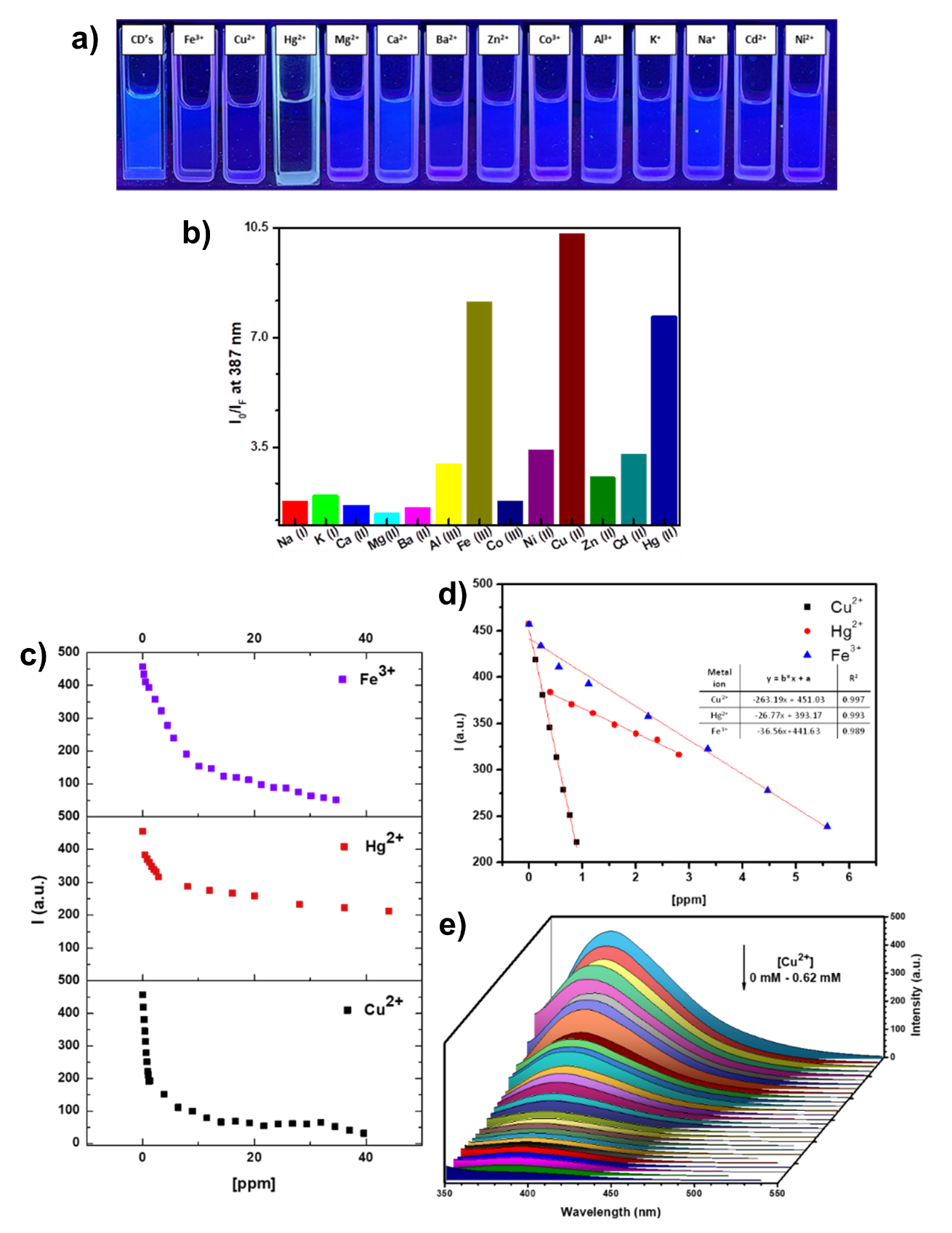


**Fig. S3. (**a) Metal binding test (MBT) for CDs (0.016 ppm) and (b) the quenching of fluorescence in aqueous solutions for CDs (0.016 ppm) at pH = 7.0 upon the addition of different metal ions ([M]final = 20 μM). (c) Fluorescence intensity vs. concentration of metal ions; (d) Linear relationship between the intensity and the concentration (in the range of 0−14 μM); (e) Spectral titration for CDs (0.016 ppm) with Cu^2+^ (0 mM to 0.62 mM).


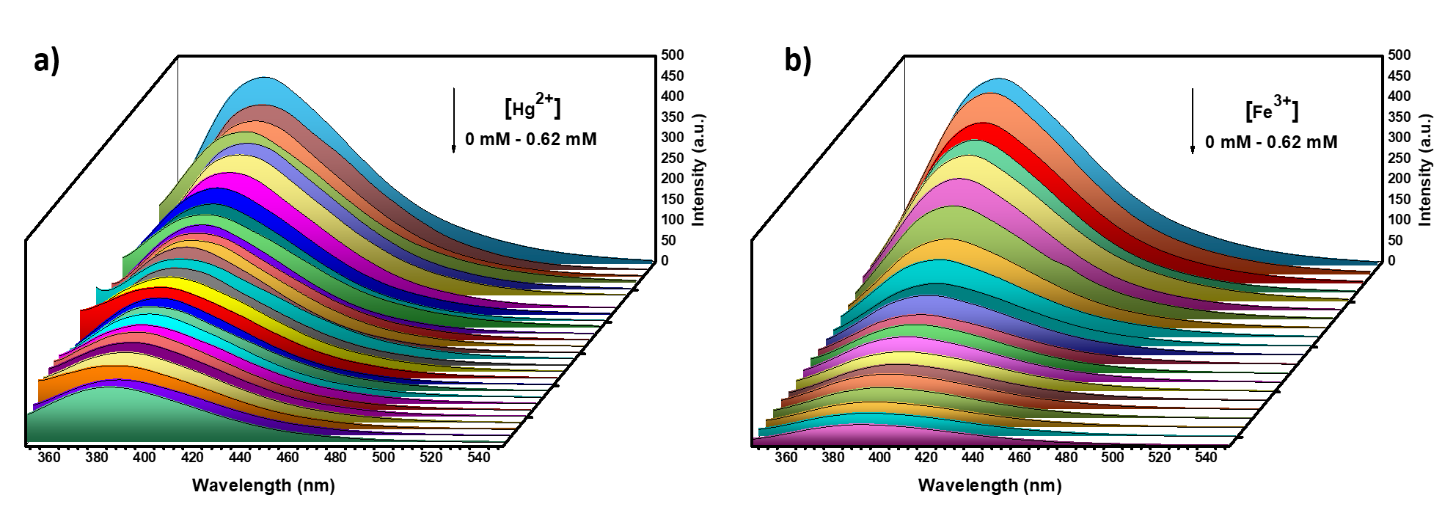


**Fig. S4.** Fluorescence titration spectrum for CDS (25 mg/ 3.0 mL) with; (a) Hg^2+^ (0 mM to 0.62 mM), (b) Fe^3+^ (0 mM to 0.62 mM).


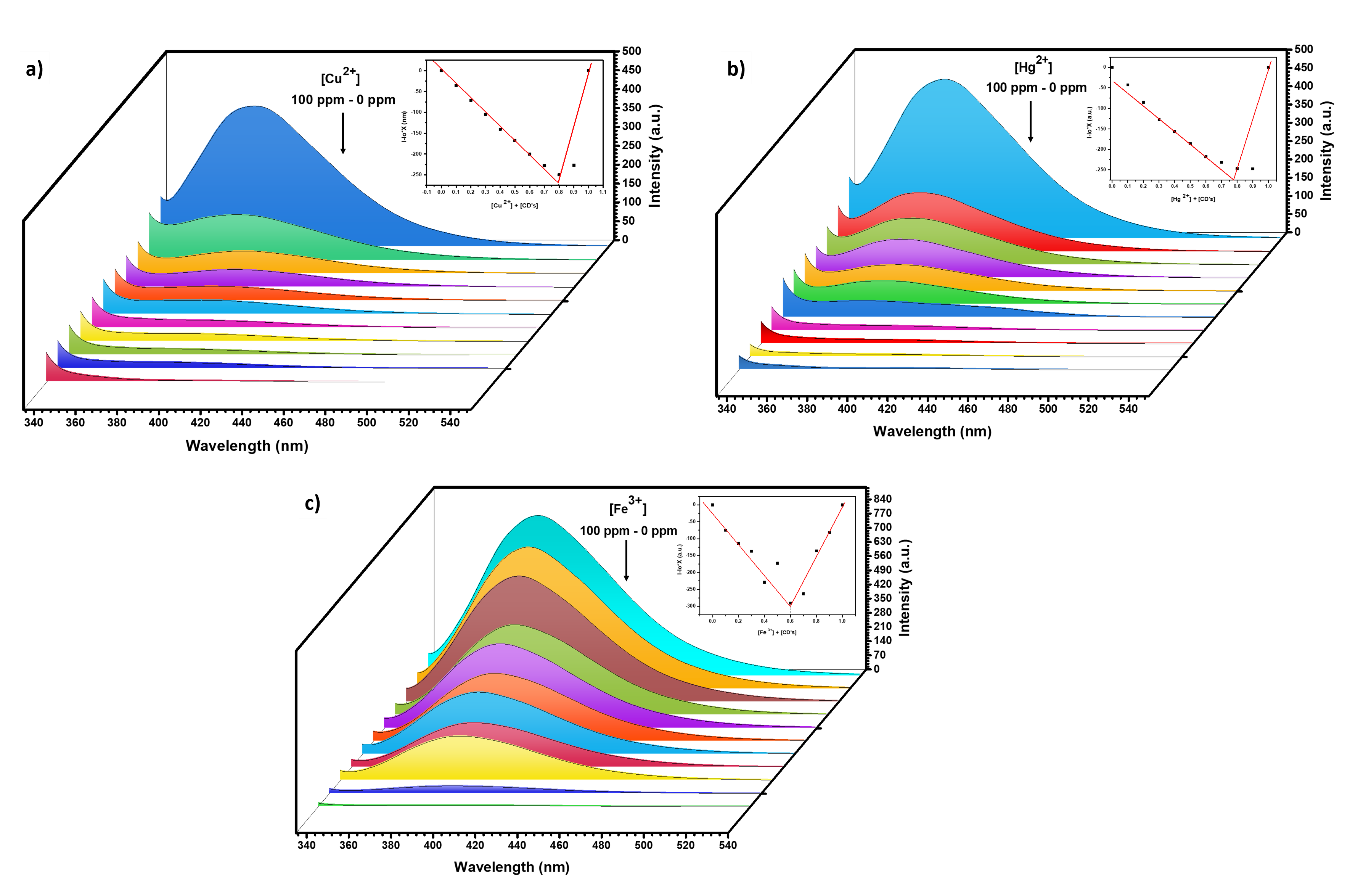


**Fig. S5.** Job’s plot; CDs vs Cu^2+^ at pH. 7; (b) for CDs vs Hg^2+^; (c) for CDs vs Fe^3+^.

**
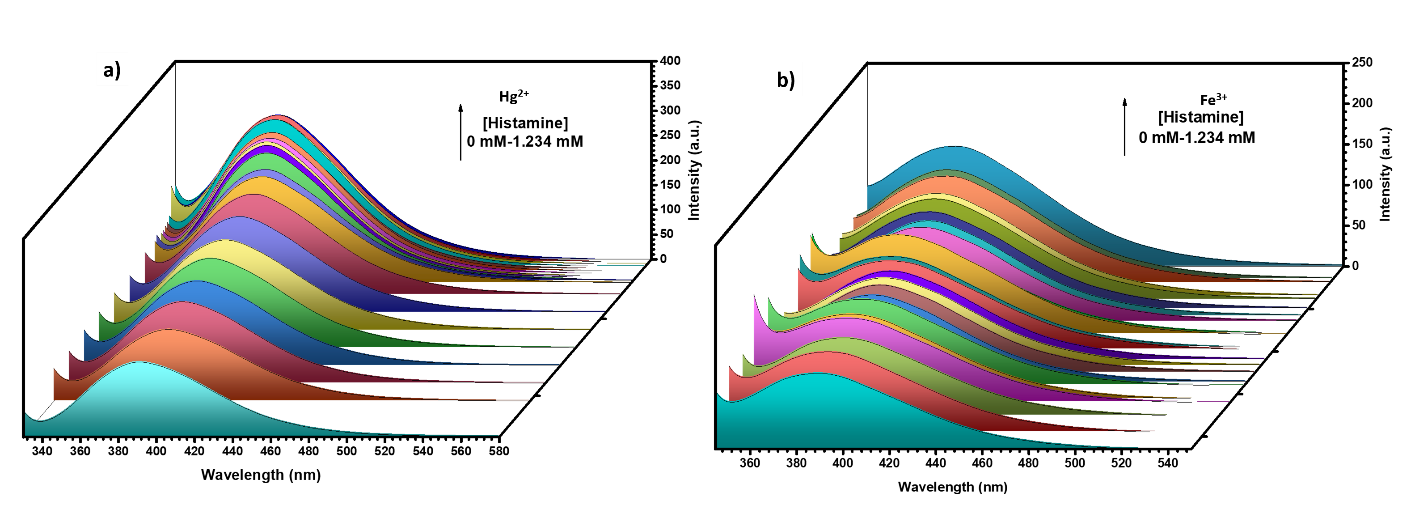
**

**Fig. S6.** Fluorescence spectra of histamine (0-1.234 mM) in the presence of; (a) Hg^2+^ and CDs, (b) Fe^3+^ and CDs.


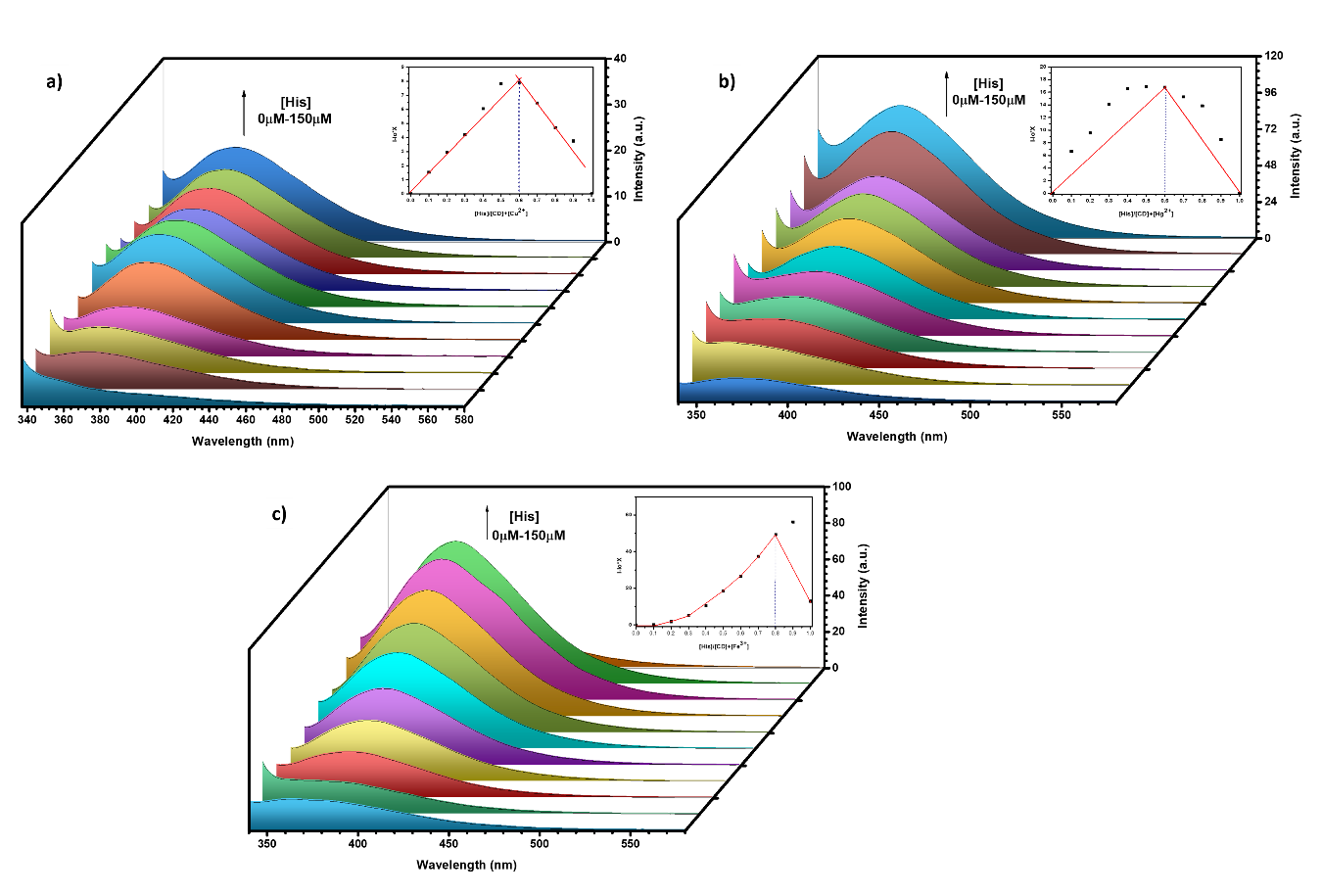


**Fig. S7.** Job’s plot at pH 7; His/CDs+Cu^2+^, (b) His/CDs+Fe^3+^, (c) His/CDs +Hg^2+^.





**Fig. S8** Metal binding test (MBT) for diamines (histamine, ethylendiamine, cadaverine, putrescine, spermine, spermidine, dimethyl-1,3-propanediamine and dimethylethylene-diamine), ([Diamine]final= 0.01mM) and the aqueous solution of CDs-Mn+(0.6437 ppm), the turn-on of fluorescence in aqueous solutions at pH = 7.0.

**Fig. S9.** Molecular orbital HOMO-LUMO of: a) **AR**-C-dots, b) **AR**-COOH, c) **AR**-OH, d) **AR**-NH_2_ and e) **AR**-SH at gaseous state.

**Fig. S10.** Molecular orbital HOMO-LUMO of a) **AR**-Fe, b) **AR**-COOH-Fe, c) **AR**-OH-Fe at gaseous state.

**Fig. S11.** Molecular orbital HOMO-LUMO of a) **AR**-Cu, b) **AR**-COOH-Cu, c) **AR**-NH_2_-Cu at gaseous state

**Fig. S12.** Molecular orbital HOMO-LUMO of: a) **AR**-Hg, and b) **AR**-SH-Hg at gaseous state

**(i)**

**(ii)**

**Fig. S13.** DT-DFT absorption spectra calculated for **i):** a) **AR**-Fe^3+^, **AR**-COOH-Fe, **AR**-OH-Fe; b) **AR**-Cu^2+^, **AR**-COOH-Cu, **AR**-NH_2_-Cu, and c) **AR**-Hg^2+^ and **AR**-SH-Hg, and **ii**) DOS for **AR**-Fe, **AR**-Cu, and AR-Hg complexes.

**Fig. S14a.** UV–visible spectra of C-dot(ads) (**AR**) +Fe^3+^ and (**AR**) isolated, both with a) COOH and b) OH group.

**Fig. S14b.** UV–visible spectra of C-dot(ads) (**AR**)+Cu^2+^ and (**AR**) isolated, both with a) COOH and b) NH_2_ group

**Fig. S14c.** UV–visible spectra of C-dot(ads) (**AR**) + Hg^2+^ and **AR** isolated, both with a) SH and b) S group.

**Fig. S15.** UV–visible spectra of C-dot(ads)+Fe^3+^ and C-dot.
